# Bacterial stigmasterol degradation involving radical flavin delta-24 desaturase and molybdenum-dependent C26 hydroxylase

**DOI:** 10.1016/j.jbc.2024.107243

**Published:** 2024-03-30

**Authors:** Tingyi Zhan, Christian Jacoby, Martin Jede, Bettina Knapp, Sascha Ferlaino, Andreas Günter, Friedel Drepper, Michael Müller, Stefan Weber, Matthias Boll

**Affiliations:** 1 Faculty of Biology, Department of Microbiology, University of Freiburg, Freiburg, Germany; 2 Faculty of Biology, Department of Biochemistry and Functional Proteomics, University of Freiburg, Freiburg, Germany; 3 Institute of Pharmaceutical Sciences, University of Freiburg, Freiburg, Germany; 4 Institute of Physical Chemistry, University of Freiburg, Freiburg, Germany

**Keywords:** steroid degradation, anaerobic metabolism, stigmasterol, flavin radical enzyme, desaturase, molybdenum enzyme

## Abstract

Sterols are ubiquitous membrane constituents that persist to a large extent in the environment due to their water insolubility and chemical inertness. Recently, an oxygenase-independent sterol degradation pathway was discovered in a cholesterol-grown denitrifying bacterium *Sterolibacterium* (*S*.) *denitrificans*. It achieves hydroxylation of the unactivated primary C26 of the isoprenoid side chain to an allylic alcohol via a phosphorylated intermediate in a four-step ATP-dependent enzyme cascade. However, this pathway is incompatible with the degradation of widely distributed steroids containing a double bond at C22 in the isoprenoid side chain such as the plant sterol stigmasterol. Here, we have enriched a prototypical delta-24 desaturase from *S. denitrificans*, which catalyzes the electron acceptor-dependent oxidation of the intermediate stigmast-1,4-diene-3-one to a conjugated (22,24)-diene. We suggest an α_4_β_4_ architecture of the 440 kDa enzyme, with each subunit covalently binding an flavin mononucleotide cofactor to a histidyl residue. As isolated, both flavins are present as red semiquinone radicals, which can be reduced by stigmast-1,4-diene-3-one but cannot be oxidized even with strong oxidizing agents. We propose a mechanism involving an allylic radical intermediate in which two flavin semiquinones each abstract one hydrogen atom from the substrate. The conjugated delta-22,24 moiety formed allows for the subsequent hydroxylation of the terminal C26 with water by a heterologously produced molybdenum-dependent steroid C26 dehydrogenase 2. In conclusion, the pathway elucidated for delta-22 steroids achieves oxygen-independent hydroxylation of the isoprenoid side chain by bypassing the ATP-dependent formation of a phosphorylated intermediate.

Steroids with an isoprenoid side chain attached to the sterane skeleton are ubiquitous natural organic molecules that are characterized by a low chemical reactivity and near insolubility in water. Among the steroids, the sterols contain a hydroxyl group at C3 and are important components of biological membranes. They influence membrane fluidity and permeability and are involved in many signal transduction processes and general lipid metabolism (1, 2). Cholesterol is the dominant sterol in animal membranes, from which bile acids and vitamin D are derived. Ergosterol is common in fungi (3), while β-sitosterol, stigmasterol, and campesterol are the main phytosterols (4). In addition to some variations in the sterane skeleton, sterols differ in the isoprene side chain by the presence/absence of an alkyl side chain at C24 and/or by a delta-22 double bond. Both modifications are common in mycosterols and phytosterols.

Complete biological degradation of steroids is of global importance for biomass decomposition and for the removal of bioactive contaminants and is only achieved by microorganisms (5, 6, 7). The high hydrophobicity of sterols makes them only poorly bioavailable resulting in the accumulation of steroids in surface waters and aquifers. Steroid hormones affect the sexual behavior of freshwater animals and the risk of prostate or breast cancer in humans through endocrine disruption by direct or indirect exposure (8).

The aerobic degradation pathways of sterols have been extensively studied in the last 3 decades in several bacteria, in particular cholesterol degradation in model strains of the genera *Rhodococcus, Mycobacterium* (both *Actinobacteria*) or *Comamonas* (*Betaproteobacteria*) (9, 10, 11, 12, 13). The initial steps of aerobic cholesterol degradation involve the oxidation/isomerization of ring A and the hydroxylation and oxidation of C26 of the isoprenoid side chain to a carboxylic acid catalyzed by cytochrome P450 monooxygenases (*e.g.*, CYP125) (Fig. 1, left panel). After activation to a CoA ester, the isoprenoid side chain is converted to propionyl-CoA and acetyl-CoA via modified β-oxidation reactions, resulting in the formation of the central intermediate androst-1,4-diene-3,17-dione (ADD) (14, 15, 16). While the degradation of cholesterol side chain has been studied in detail, the genes and enzymes involved in the degradation of sterols with unsaturated side chains have remained largely unknown.

**Figure 1:**
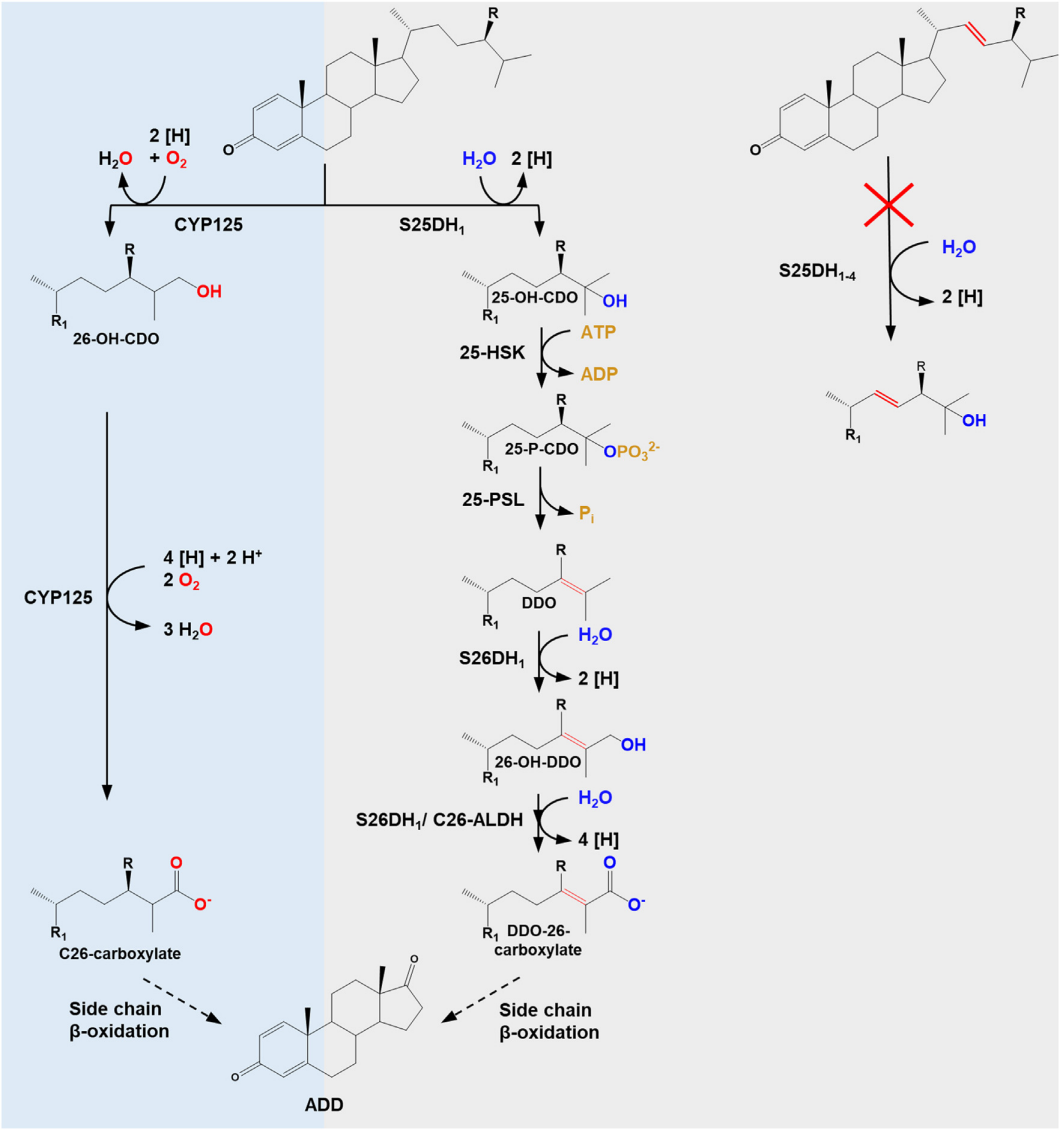
**Initial steps of steroid isoprenoid side chain degradation in bacteria.***Left panel* (*light blue background*), O_2_-dependent oxidation of primary C26 to a carboxylic acid by CYPs in aerobic bacteria; *middle panel* (*gray background*) O_2_-independent oxidation of C26 to a carboxylic acid *via* an ATP-dependent enzyme cascade in denitrifying bacteria; *right panel* (*gray background*) steroids with a Δ22 double bond are no substrates for S25DHs. R = H, methyl or ethyl; R_1_ = androst-1,4-diene-3-one-17-yl; 25-HSK, 25-hydroxysteroid kinase; 25-PSL, 25-phosphosteroid lyase; ADD, androst-1,4-diene-3,17-dione; C26-ALDH, C26-aldehyde dehydrogenase; CDO, cholest-1,4-diene-3-one; CYP125, cytochrome P450 monooxygenase; DDO, desmost-1,4-diene-3-one; S25DH_1_, steroid C25 dehydrogenase 1; S26DH_1_, steroid C26 dehydrogenase 1.

In the absence of molecular oxygen as a cosubstrate, a fundamentally different enzymology is required for the full oxidation of steroids to CO_2_. To date, only a few bacterial strains are known that are able to grow with sterols as the sole carbon source under denitrifying conditions, and insights into the enzymatic steps involved have come mainly from studies of cholesterol degradation in *Sterolibacterium* (*S.*) *denitrificans* strain Chol-1S^T^ (17). Similar to aerobic cholesterol degradation, the pathway is initiated by an isomerization and oxidation of ring A to cholest-1,4-diene-3-one (CDO) (18, 19). However, the next steps involved in the activation of the isoprenoid side chain are fundamentally different in aerobic and anaerobic bacteria (Fig. 1).

In the absence of molecular oxygen, CDO is hydroxylated at the tertiary C25 with water as hydroxylating agent by the αβγ-heterotrimeric molybdenum (Mo)-dependent steroid C25 dehydrogenase 1 (S25DH_1_) (Fig. 1, middle panel) (20, 21, 22). This enzyme belongs to the type II DMSO reductase family of metalloprotein cofactor-containing enzymes and contains a molybdenum-bis-metalloprotein guanine dinucleotide cofactor in its active site. A mechanism involving a hydride transfer from the substrate to a Mo(VI)=O species, yielding a relatively stable tertiary carbocation has been proposed (23). The latter intermediate then abstracts a hydroxyl group from the Mo(VI)-OH formed to give the tertiary C25 alcohol. This intermediate is then phosphorylated by ATP-dependent 25-hydroxysteroid kinase to 25-phospho-CDO (24), from which the phosphate is eliminated by a putative 25-phosphosteroid lyase yielding the Δ24 subterminal alkene desmost-1,4-diene-3-one (DDO) (25). The formation of an allylic double bond at C24 allows for the second water-dependent hydroxylation of the primary C26 to the allylic C26-alcohol by the Mo-dependent steroid C26 dehydrogenase 1 (S26DH_1_) (25). S26DH_1_ shares high amino acid sequence identity with other S25DHs, suggesting a similar reaction mechanism. During S26DH catalysis, the abstraction of the hydride from the primary C26 is facilitated by the formation of an allylic carbocation intermediate, which is enabled by the Δ24 double bond formed in the preceding steps. The same S26DH enzyme catalyzes the further oxidation of the allylic alcohol (26-OH-DDO) to the corresponding aldehyde (DDO-26-al), followed by oxidation of the latter to DDO-26-carboxylate by a putative C26 aldehyde dehydrogenase (25). Further degradation proceeds via similar β-oxidation reaction sequences as in the aerobic degradation of cholesterol to the common intermediate androst-1,4-diene-3,17-dione (26, 27).

In addition to cholesterol, *S. denitrificans* is known to use a range of phyto- and mycosterols as carbon and energy sources under denitrifying conditions (26). These include β-sitosterol with a saturated isoprenoid side chain and an ethyl substituent at C24 and sterols with both, a Δ22 double bond and a C24 alkyl substituent such as stigmasterol or ergosterol. The presence of genes encoding four variants of Mo-containing S25DHs (S25DH_1-4_) and three encoding putative S26DHs (S26DH_1–3_) in *S. denitrificans* suggests that they might be involved in the hydroxylation of tertiary C25 and allylic C26 intermediates during the degradation of the phytosterols and mycosterols (22). Notably, these S25DH/S26DH-like gene products are produced at different levels during growth with individual steroids. Indeed, S25DH_4_ was induced during growth with β-sitosterol, and the enriched enzyme showed a preference for the β-sitost-4-en-3-one intermediate (22, 26). However, none of the four S25DH_1-4_ present in *S. denitrificans* accepted stigmast-1,4-diene-3-one (SDO) or ergosterol as substrate, although they serve as carbon and energy sources during cultivation (Fig. 1, right panel) (22). It has been proposed that the presence of the Δ22 double bond sterically prevents a promiscuous binding to S25DH_1–4_.

In the present study, we investigated the unknown degradation of steroids with unsaturated side chains in the model organism *S. denitrificans*. Possible scenarios include the reduction to a saturated isoprenoid side chain, which could then serve as a substrate for S25DH_1_. However, such a reduction of an isolated, nonactivated double bond is difficult to achieve under anoxic conditions. As an alternative, a second double bond could be introduced in conjugation with the one already present at C22. We show that SDO is oxidized to a Δ1,4,22,24-tetraene catalyzed by a previously unknown flavin-dependent desaturase, most likely by a radical-based mechanism. The conjugated Δ22,24 moiety formed then allows for the water-dependent hydroxylation to the allylic alcohol by S26DH_2_. This pathway bypasses the ATP-dependent enzyme cascade used to degrade steroids with saturated isoprenoid side chains.

## Results

### SDO oxidizing activity in extracts of stigmasterol-grown cells

**Figure 2:**
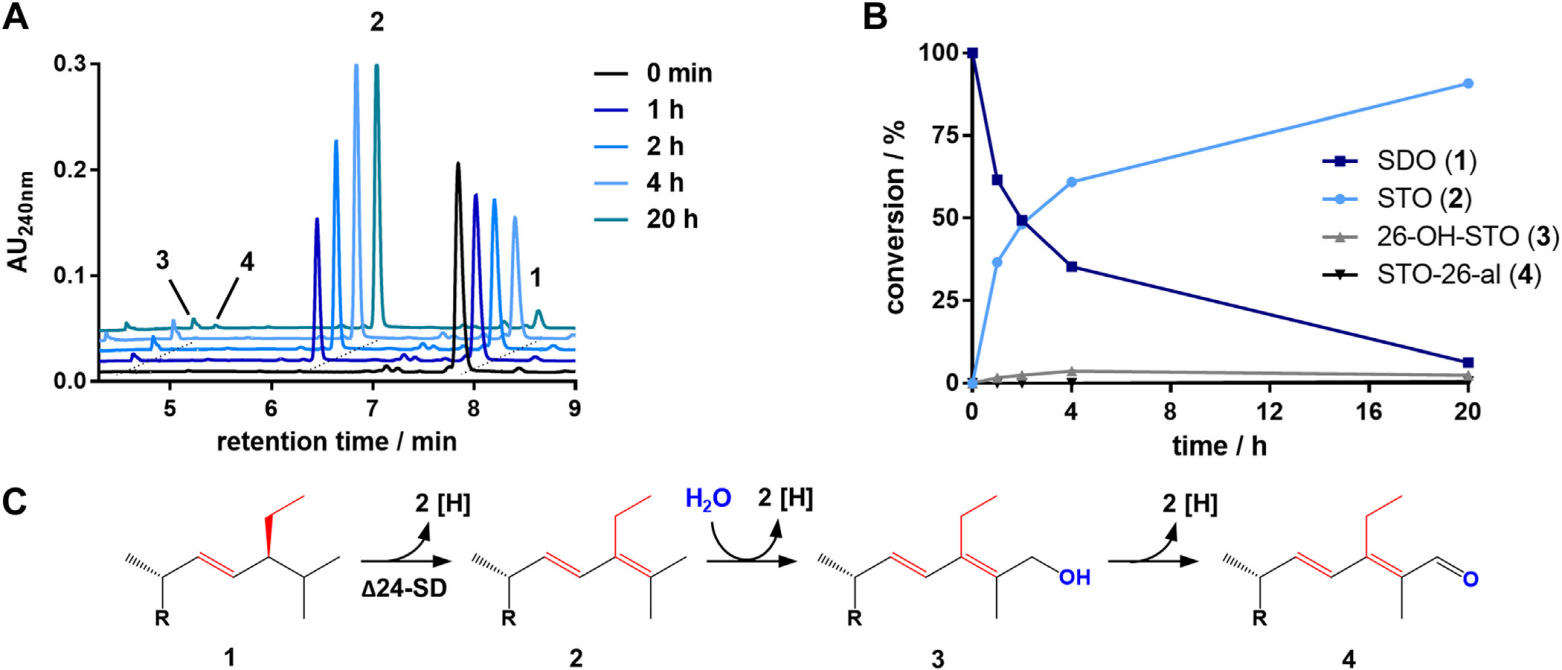
**Conversion of stigmast-1,4-diene-3-one (SDO) to oxidized products using soluble protein extract from *Sterolibacterium denitrificans* and DCPIP as an artificial electron acceptor.***A*, UPLC elution chromatogram showing the time-dependent conversion of 0.5 mM SDO **1** to STO **2** and other minor products **3** and **4** by the soluble protein fraction of *S. denitrificans* extracts. *B*, representative plot showing the quantitative analysis of the UPLC-based assay. *C*, proposed reaction sequence from SDO **1***via* STO **2** to 26-OH-STO **3** and STO-26-al **4**. DCPIP, 2,6-dichlorophenolindophenol; UPLC, ultra-performance liquid chromatography; STO, stigmasta-1,4,24-triene-3-one.

The unknown initial steps of stigmasterol side chain degradation were investigated in cell-free extracts of *S. denitrificans* cells grown with stigmasterol and nitrate as sole energy and carbon sources. Cultivation in a 200-l fermenter yielded 160 g cells (wet mass) with a doubling time of around 60 h (for growth curve, Fig. S1). The proposed SDO intermediate with the typical 1,4-diene-3-one structure in ring A was enzymatically synthesized from stigmasterol using AcmB and cholesterol oxidase (25). Assays for SDO conversion contained 6% (w/v) 2-hydroxypropyl-β-cyclodextrin (HPCD) as a solubilizing agent. In agreement with previous observations (22), no K_3_[Fe(CN)_6_]-dependent hydroxylation of 0.5 mM SDO to 25-OH-SDO was observed. Moreover, no reduction of the C22 double bond was detected in the presence of strong reducing agents such as Ti(III)-citrate or sodium dithionite (5 mM each). Instead, using 2,6-dichlorophenolindophenol (DCPIP) as electron acceptor, the time- and protein-dependent conversion of SDO to polar products was observed during ultra-performance liquid chromatography (UPLC) analysis of samples taken at different time points (Fig. 2, *A* and *B*). After ultracentrifugation, the activity was completely found in the soluble fraction. Electrospray ionization quadrupole time-of-flight mass spectrometry analysis identified the major product **2** as a compound with a m/z value of approximately 2 Da lower than that of SDO suggesting that it represents a dehydrogenated product (for calculated and experimentally determined masses in this work, see Table S1). On prolonged incubation, other minor polar products were observed with m/z values consistent with a hydroxylation of the product to the C26 alcohol **3** and the dehydrogenation of the latter to the aldehyde **4**.

Products **2** and **3** were purified by preparative HPLC and identified as stigmasta-1,4,24-triene-3-one (STO) and (24*E*)-26-hydroxy-STO (26-OH-STO) by ^1^H, ^13^C, and 2D NMR analyses (for NMR spectra, Figs. S2–S6), confirming the reaction sequence shown in Figure 2*C*.

### Enrichment of Δ24-steroid desaturase

**Figure 3:**
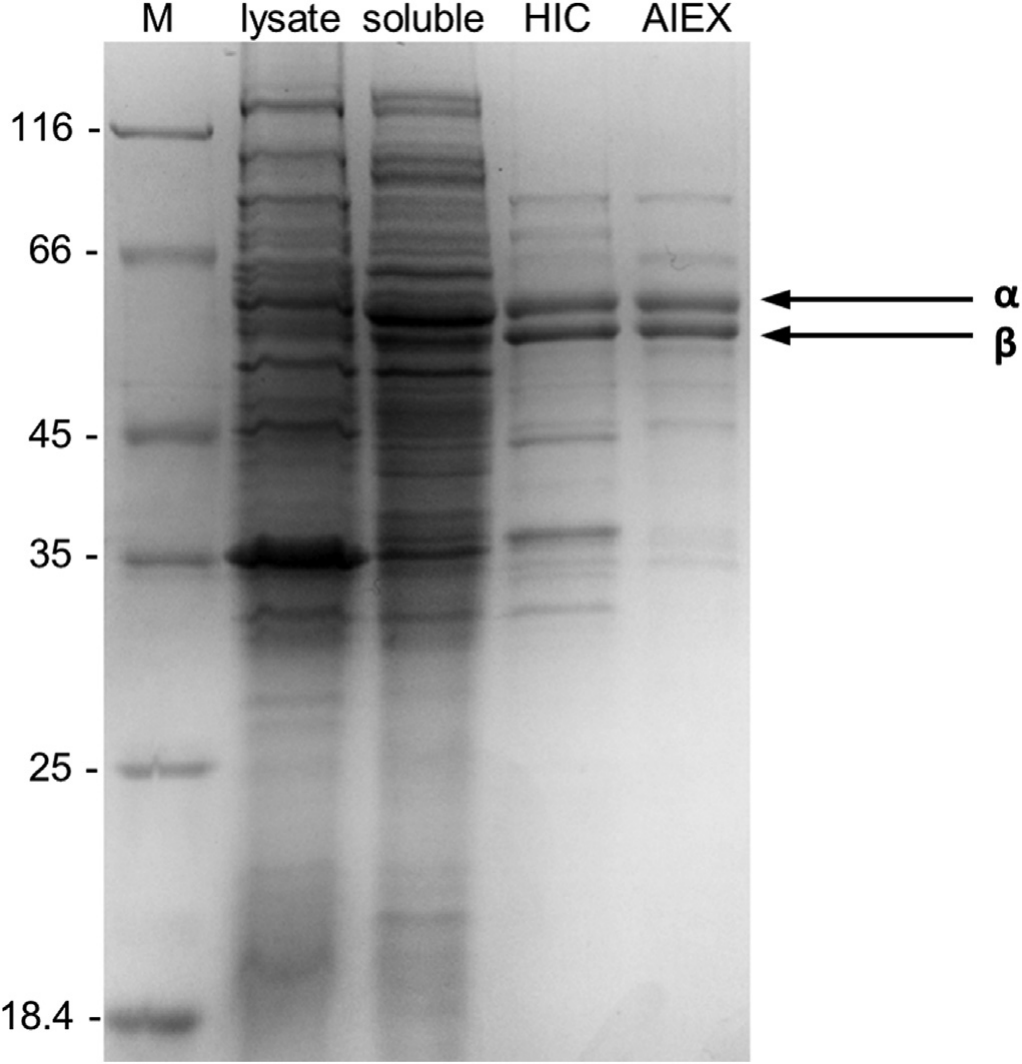
**SDS-PAGE analysis of Δ24-steroid desaturase during enrichment from WT *Sterolibacterium. denitrificans*.** Lysate: 20 μg protein after cell disruption, soluble: soluble proteins after ultracentrifugation at 45,000 rpm, HIC: 10 μg protein after Butyl-S FF chromatography, AIEX: 6 μg proteins after HiTrap Capto Q chromatography, M: molecular size marker (kDa).

To identify the unknown enzyme(s) involved in the desaturation of SDO to STO, enrichment was performed from soluble cell-free extracts of *S. denitrificans* grown on stigmasterol and nitrate. Activity was monitored using the UPLC-based enzymatic assay for the conversion of SDO to STO during each chromatographic enrichment step. Enrichment of the desaturase activity by Butyl-S Sepharose 6 Fast Flow (Butyl-S FF) hydrophobic interaction chromatography followed by HiTrap Capto Q anion-exchange chromatography yielded two major protein bands at approximately 60 kDa by SDS-PAGE analysis (Fig. 3). The desaturase, hereafter referred to as Δ24-steroid desaturase (Δ24-SD), was enriched to a final yield of approximately 60% with an enrichment factor of 22 (Table 1). The molecular weight (MW) of Δ24-SD was determined by size-exclusion chromatography (Superose 6 Increase 10/300 GL) to be approximately 440 kDa, which is most consistent with a heterotetrameric α_4_β_4_ composition (calculated mass ≈ 480 kDa) (for measured molecular weight, Fig. S7).

**Table 1:** Enrichment of Δ24-SD from 25 g (wet mass) *Sterolibacterium denitrificans* cells grown with stigmasterol and nitrate

Fractions	Protein [mg]	Specific activity [nmol min^–1^ mg^–1^]	Yield [%]	Enrichment (fold)
Soluble cell extract	1260	2.1		
Butyl-S FF	13.6	38	20	18
HiTrap Capto	7.7	47	14	22

The enriched protein bands migrating at approximately 60 kDa were excised, digested with trypsin, and analyzed by electrospray ionization quadrupole time-of-flight mass spectrometry analysis. The encoding genes identified are located next to each other in the genome of *S. denitrificans*, and are assigned the two structural α- (63 kDa, accession number WP_067170564) and β- (57 kDa, accession number WP_067170561) subunits of Δ24-SD (for amino acid sequence identities see Table S2). The function of the enriched enzyme was confirmed by using the established DCPIP-dependent, UPLC-based activity assay. Conversion of more than 90% of SDO to STO was achieved within 24 h with an initial specific activity of approximately 47 nmol min^–1^ mg^–1^ (for SDO conversion with enriched Δ24-SD, Fig. S8). No formation of 26-OH-STO and STO-26-al was observed, suggesting that Δ24-SD does not catalyze the further oxidation of the alcohol to the aldehyde and that the enzyme(s) involved in their formation was removed during the enrichment of Δ24-SD.

### Catalytic properties

The catalytic properties of Δ24-SD were determined using the UPLC-based enzymatic assay at substrate concentrations up to 2 mM SDO in the presence of 6% (w/v) HPCD. A fit of the data obtained to a Michaelis–Menten curve gave a *V*_max_ of 42.5 ± 1.5 nmol min^–1^ mg^–1^ and a *K*_m_ of 51.5 ± 8.2 μM (mean values ± standard deviation of three independent measurements) (for Michaelis–Menten curve, Fig. S9). The apparent affinity of Δ24-SD to its substrate was significantly higher than reported for other characterized side chain-containing steroid degrading enzymes [*e.g.*, *K*_m_ of 390 ± 8 μM for heterologously produced S25DH_1_ (22) and *K*_m_ of 123 ± 25 μM for heterologously produced S26DH_1_ (25)]. Commercially available steroids with isoprenoid side chain modifications were tested as alternative substrates, including ergosterol, brassicast-1,4-diene-3-one (with a C22 double bond and a methyl group at C24 in [*R*] configuration), fucost-1,4-diene-3-one (containing an ethylidene group at C24) and β-sitost-1,4-diene-3-one (with an (*R*)-configured ethyl group at C24) (for structures see Table S3). None of these substrates were converted by Δ24-SD (<1% conversion compared to SDO), suggesting that Δ24-SD is only active with substrates containing a C22 double bond, and that alkyl substituents at C24 are only accepted in (*S*) but not in (*R*) configuration.

### Phylogenetic analysis reveals similarities to limonene dehydrogenase

**Figure 4:**
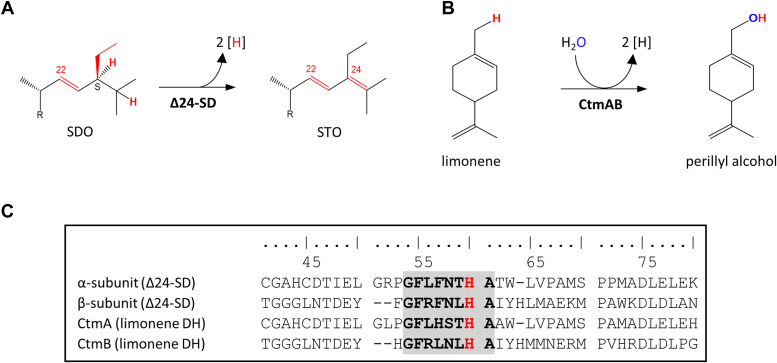
**Reactions catalyzed and amino acid sequences involved in covalent flavin binding of Δ24-SD and limonene dehydrogenase.***A*, Δ24-SD reaction; *B*, limonene dehydrogenase (DH) reaction; *C*, amino acid sequences involved in covalent flavin cofactor binding in the two subunits of Δ24-SD and limonene dehydrogenase CtmAB. The conserved histidine to which the flavin is covalently linked is highlighted in *red*; the conserved sequence region around this histidine is shown in *gray*.

Amino acid sequence comparison between the α- and β-subunit of Δ24-SD revealed 26% amino acid sequence identity. Using BLAST, both subunits of Δ24-SD showed highest amino acid sequence similarities to uncharacterized putative NAD(P)/FAD-dependent oxidoreductases from closely related steroid-degrading *Sterolibacterium* sp. as well as from species of the *Pseudomonadales*, *Halieaceae*, *Acidimicrobiales*, or *Deltaproteobacteria* (for phylogenetic trees, Figs. S10 and S11). The highest amino acid sequence identities to a biochemically characterized enzyme were to the CtmA (α-subunit, 34%) and CtmB (β-subunit, 38%) subunits of the heterodimeric limonene dehydrogenase (DH) CtmAB from the *Betaproteobacterium* *Castellaniella defragrans* (28). This flavoenzyme catalyzes the hydroxylation of the allylic methyl group of limonene to perillyl alcohol (Fig. 4*B*). Both, Δ24-SD and limonene DH have in common is that they oxidize carbon atoms at an α,β-unsaturated position. While Δ24-SD catalyzes a dehydrogenation reaction (Fig. 4*A*), limonene dehydrogenase uses water for a hydroxylation reaction (Fig. 4*B*). The limonene dehydrogenation reaction is comparable to that of S26DHs, but is achieved with a flavin rather than with a molybdopterin cofactor.

### Covalently bound FMN cofactors

The enriched Δ24-SD showed a dark yellow color, which is characteristic for flavoenzymes, but also for iron-containing enzymes. Therefore, the iron content of Δ24-SD was analyzed according to Lovenberg using *o*-phenanthroline as a chelating agent (29). Less than 0.05 Fe per αβ dimer was found. This finding is in agreement with limonene DH CtmAB, which does not contain iron-sulfur clusters. The flavin content of Δ24-SD was then analyzed by extraction of the flavin cofactor by acid denaturation. After centrifugation, the denatured protein pellet retained an yellow color, while the supernatant remained colorless. This result indicated that the flavin cofactors were covalently bound to Δ24-SD. Additionally, we searched for flavinylated peptides from Δ24-SD by liquid chromatography-tandem mass spectrometry analysis after tryptic digestion. Indeed, peptides from both, the α- and β-subunits of Δ24-SD were identified that contained flavin mononucleotide (FMN) modifications in both subunits (for MS/MS spectra, Fig. S12) at a histidyl residue that is part of a conserved consensus sequence near the N terminus involved in covalent FMN binding, most likely *via* the 8-methyl group of the cofactor (Fig. 4*C*). Notably, this consensus sequence (G-F-L/R-X-N/S-T/L-H-A) is also present in both subunits of limonene DH CtmAB.

### UV/visible absorption and EPR spectroscopic analyses favor a radical-based mechanism

**Figure 5:**
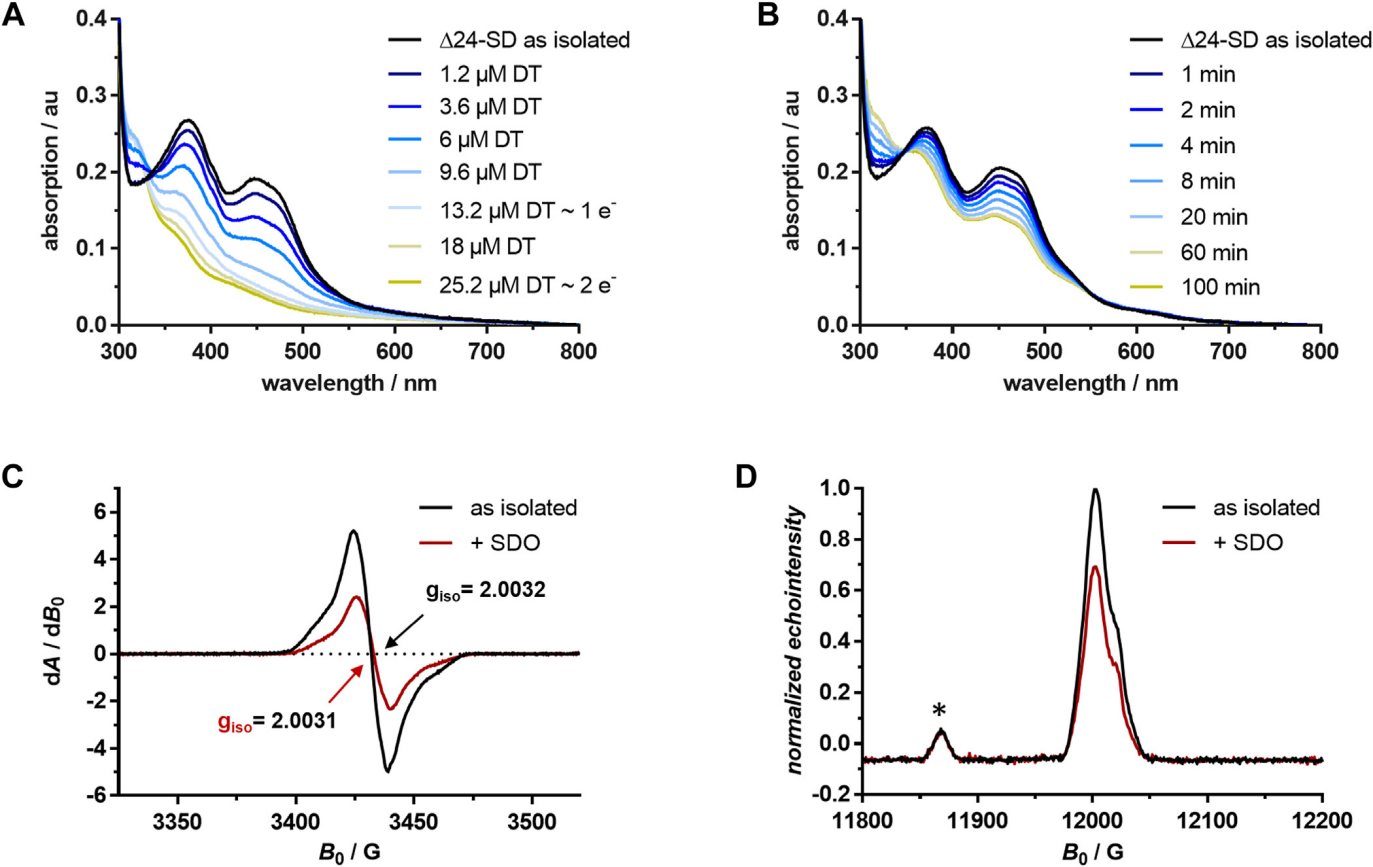
**Spectroscopic analysis of Δ24-SD during the reduction by sodium dithionite (DT) and by SDO.***A*, UV/visible absorption spectra after stepwise addition of increasing equivalents of DT to 25 μM Δ24-SD. *B*, UV/visible absorption spectra taken at different time points after addition of 25 μM SDO to 25 μM Δ24-SD. *C*, continuous-wave (cw) EPR spectra of Δ24-SD (15.5 mg ml^–1^) as isolated (*black*) and with stoichiometric equivalents of SDO (*red*) at X-Band frequency (9.63 GHz). *D*, field-sweep echo-detected (FSED) EPR spectra of Δ24-SD as isolated (*black*) and in the presence of stoichiometric equivalent amount of SDO (*red*) at Q-band frequency (33.69 GHz). Echo intensities were normalized to the as isolated sample. The small signal marked with an *asterisk* (∗) is assigned to a background signal from the resonator. UV/visible spectra are baseline-corrected at 800 nm. Au, arbitrary units; *B*_0_, magnetic field; d*A*/d*B*_0_, first derivative of the absorption line obtained by field modulation; EPR, electron paramagnetic resonance; G, gauss; SDO, stigmast-1,4-diene-3-one.

The properties of the covalently bound FMN cofactors were analyzed by UV/visible absorption spectroscopy. The absorption spectrum of Δ24-SD as isolated in the oxidized state showed maxima at 377 nm and 450 nm with a shape that is characteristic of the red anionic semiquinone (SQ) radical state of flavins (Fig. 5*A*). On stepwise titration with the two-electron donor sodium dithionite (DT) at pH 7.5 under anaerobic conditions, both peaks gradually decreased with virtually no change in shape. Complete reduction of 25 μM Δ24-SD was achieved at 25 μM DT, indicating that the SQ states of both FMN cofactors were reduced by one electron each. The SQ state of both flavins could not be oxidized in air (incubation for 1 h with stirring), H_2_O_2,_ or the artificial electron acceptor DCPIP (both in excess of the protein concentration) (Fig. 5*A*). This finding suggests that both FMNs only switch between the SQ and fully reduced state during catalysis. Surprisingly, only half of the anionic red SQ is reduced upon addition of stoichiometric amounts of the two-electron donor SDO (25 μM) (Fig. 5*B*). Even in the presence of a 5-fold excess of SDO, no further reduction was observed.

Continuous wave (CW) and magnetic-field sweep echo-detected electron paramagnetic resonance (EPR) spectra of Δ24-SD as isolated sample showed a single radical species assigned to the SQ state of both flavins (Fig. 5, *C* and *D*). The *g*-principal values of the flavin radical signal as isolated were obtained from spectral simulations: *g*_x_ = 2.0046, *g*_y_ = 2.0035, *g*_z_ = 2.0014 giving *g*_iso_ = 2.0032. Those for the sample in the presence of substrate were *g*_*x*_ = 2.0039, *g*_y_ = 2.0035, *g*_z_ = 2.0019 giving *g*_iso_ = 2.0031 (for EPR simulations, Fig. S13). The peak-to-peak linewidth of 14 G as well as the g values are characteristic for anionic flavin SQs (30). In full agreement with UV/visible absorption spectroscopy, the addition of stoichiometric amounts of SDO decreased the signal to about 50% of its initial intensity but did not change its shape, ruling out the formation of a stable substrate-based radical that should show a markedly altered EPR signal. Again, an excess of SDO did not further reduce the radical signal, suggesting that the reduction of only 50% of the flavins was not due to thermodynamic reasons.

### Oxidation of SDO by molybdenum dependent S26DH2

**Figure 6:**
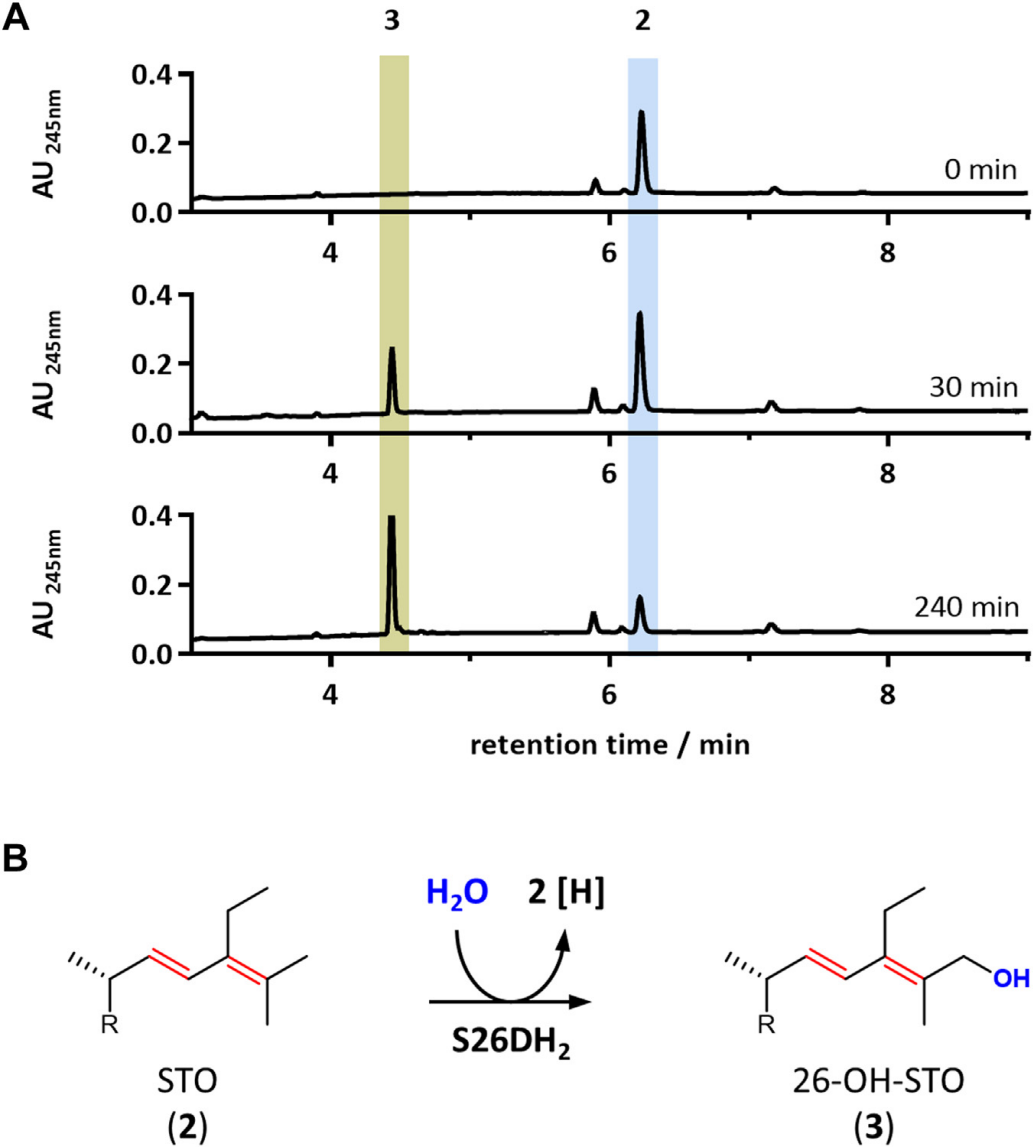
**Conversion of stigmasta-1,4,24-triene-3-one (STO) using cell extracts from *Thauera aromatica* cells that heterologously produce S26DH**_**2**_**from *Sterolibacterium denitrificans*.***A*, UPLC elution chromatogram showing the time-dependent conversion of STO (2) to 26-OH-STO (3); *B*, reaction catalyzed by S26DH_2_. S26DH, steroid C26 dehydrogenase; STO, stigmasta-1,4,24-triene-3-one; UPLC, ultra-performance liquid chromatography.

The *S. denitrificans* genome contains three gene clusters encoding the putative αβγ-subunits of related Mo-dependent S26DHs (22). One of these (WP_154715926-8) catalyzes the water-dependent hydroxylation of the allylic C26 methyl group of the cholesterol degradation intermediate DDO to the corresponding allylic alcohol. After a second hydroxylation by this enzyme, the corresponding C26 aldehyde is formed. Based on this finding, this enzyme was referred to as S26DH_1_ (25). Here, we tested the possibility that one of the three related S26DH_1-3_ is involved in the degradation of stigmasterol in *S. denitrificans* by catalyzing the hydroxylation of STO to a C26 alcohol and, presumably, its further oxidation to the C26 aldehyde. For this purpose, the three genes encoding each of the three enzymes were heterologously produced in *Thauera* (*T.*) *aromatica* and the conversion of STO to 26-OH-STO and the corresponding 26-aldehyde was assayed in cell extracts producing each S26DH. Extracts of *T. aromatica* producing S26DH_2_ (WP_154715930-32) did indeed catalyze the conversion of STO to 26-OH-STO (Fig. 6), whereas extracts producing S26DH_1_ (WP_154715926-8) or S26DH_3_ (WP_154716403-5) showed negligible activity with STO (<2% of extracts producing S26DH_1_ or S26DH_2_). The 26-OH-STO product of S26DH_2_ was only very slowly converted to the C26 aldehyde, suggesting that an additional alcohol dehydrogenase is required for the oxidation of 26-OH-STO to STO-26-al.

## Discussion

**Figure 7:**
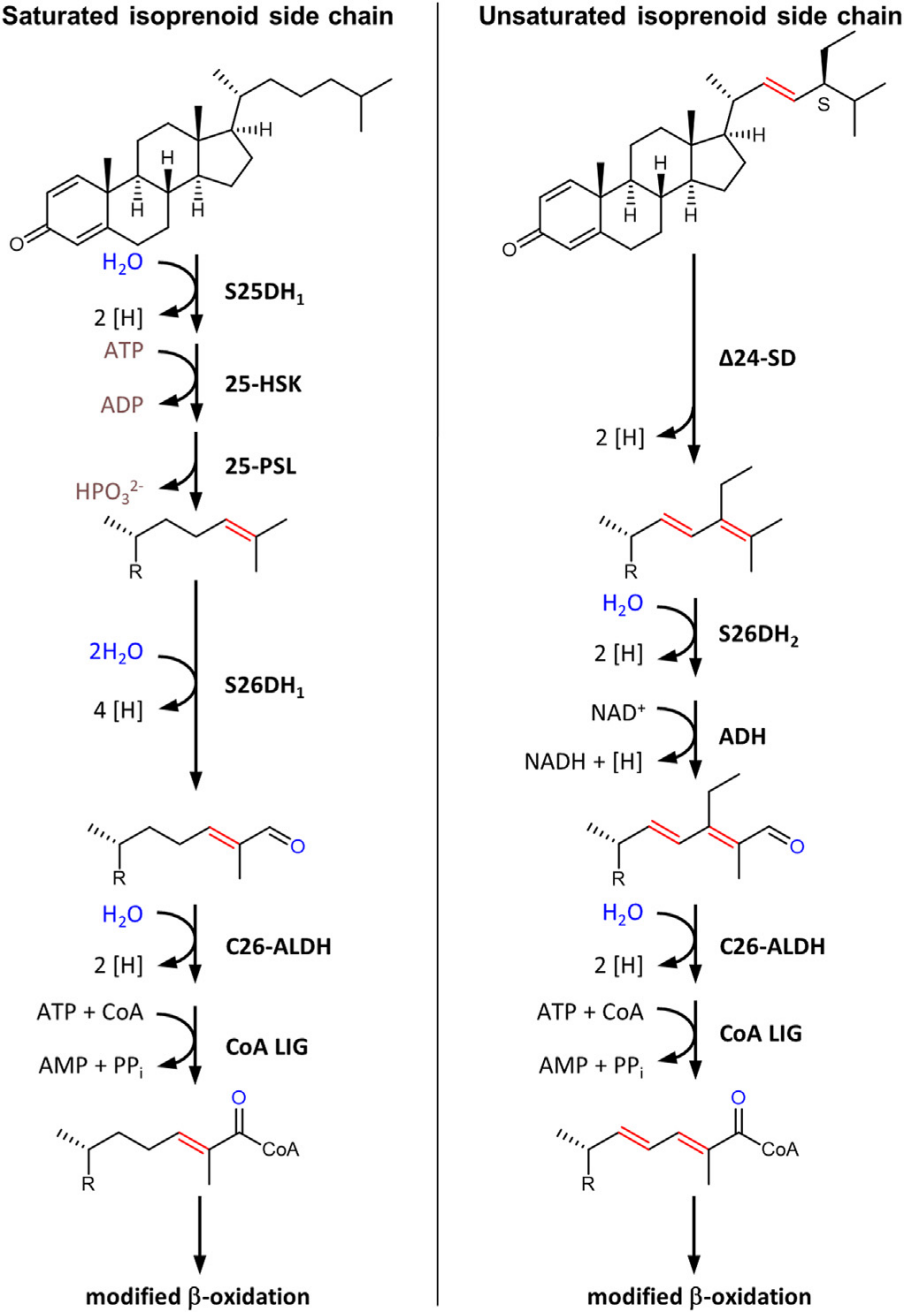
**Degradation of sterols with saturated (*left panel*) and unsaturated (*right panel*) isoprenoid side chains in anaerobic bacteria.** With unsaturated isoprenoid side chains, the ATP-dependent three-enzyme cascade involving S25DH_1_, 25-HSK and 25-PSL, shown in detail in Figure 1, can be bypassed by Δ24-SD. Note, that S26DH_1_ catalyzes both, the hydroxylation and oxidation of DDO to the C26 aldehyde, whereas S26DH_2_ catalyzes only the formation of the allylic alcohol from STO, thus requiring an additional alcohol dehydrogenase during stigmasterol degradation. 25-HSK, 25-hydroxysteroid kinase; 25-PSL, 25-phosphosteroid lyase; ADH, alcohol dehydrogenase; C26-ALDH, C26 aldehyde dehydrogenase; CoA LIG, CoA-dependent ligase; DDO, desmost-1,4-diene-3-one; S26DH, steroid C26 dehydrogenase; STO, stigmasta-1,4,24-triene-3-one.

In this work, the previously unknown genes and enzymes involved in the degradation of sterols with an unsaturated isoprenoid side chain was elucidated using stigmasterol degradation in *S. denitrificans* as model system. In contrast to the degradation pathways of steroids with saturated side chains such as cholesterol, it bypasses the ATP-dependent enzyme cascade that proceeds via a C25 tertiary phosphoester intermediate. The latter allows for the formation of a subterminal alkene by phosphate elimination, which can then be hydroxylated at the allylic C26 position, catalyzed by a specific molybdenum-dependent S26DH (24, 25). In the case of steroid substrates with a preexisting Δ22 double bond, this allylic position can be directly formed in a single step by the newly identified Δ24-SD (Fig. 7).

The subsequent water-dependent hydroxylation at C26 is then catalyzed by Mo-dependent S26DH_2_. While S26DH_1_ appears to be specific for Δ24 monoenes formed during the degradation of cholesterol with a saturated side chain, S26DH_2_ is involved in the hydroxylation of Δ22,24 diene intermediates that occur during the degradation of sterols with unsaturated isoprenoid side chains. Oxidation to the carboxylic acid and the activation to the corresponding thioester is likely to be catalyzed by specific enzymes involved in the degradation of Δ24 mono- and Δ22,24 diene intermediates. Both pathways will converge on the common C22-CoA thioester intermediate after a first modified β-oxidation cycle.

**Figure 8:**
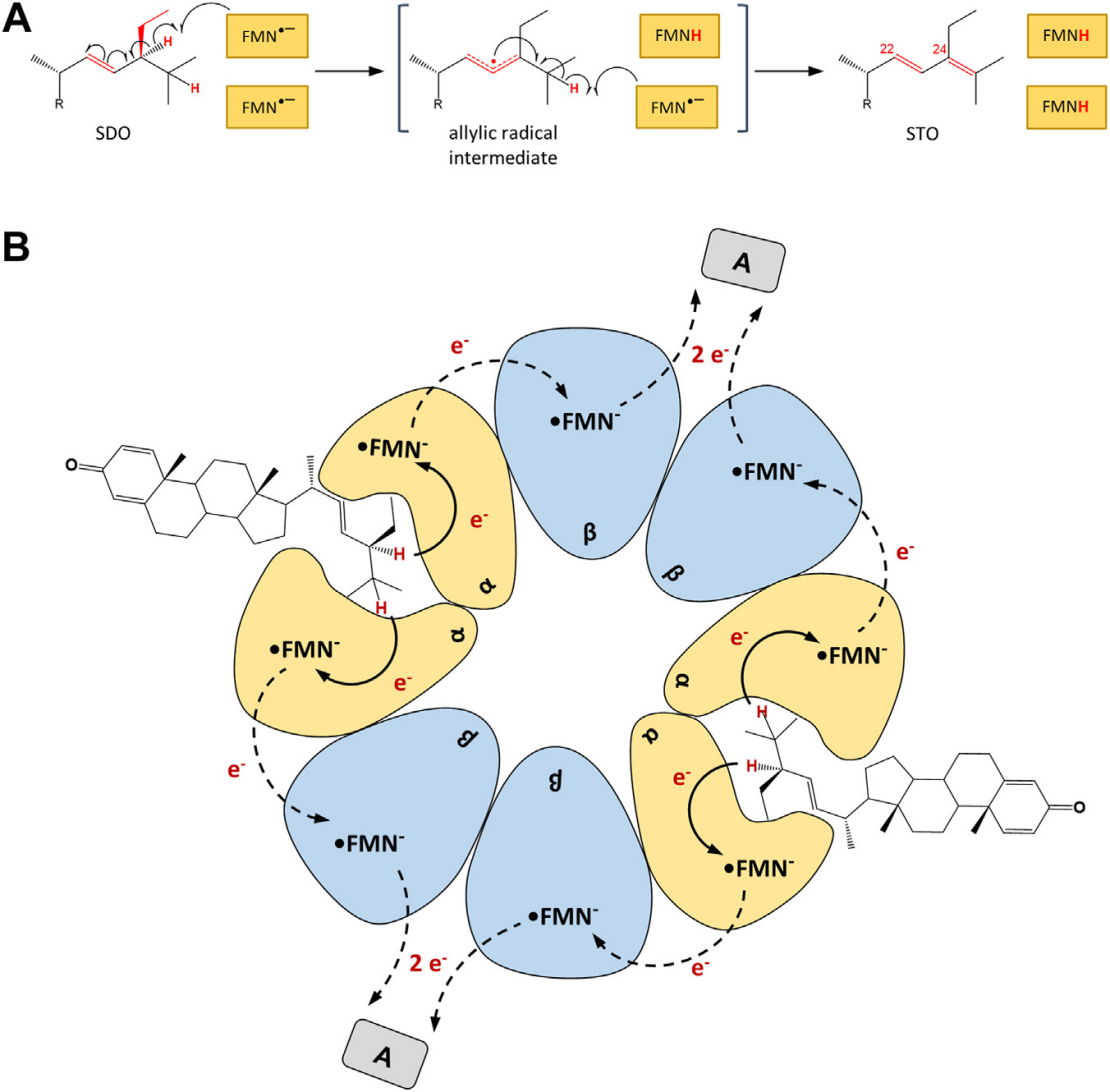
**Proposed radical mechanism of Δ24-SD and model of the subunit architecture and substrate binding.***A*, proposed mechanism in which two flavin SQs each abstract one hydrogen atom. *B*, model of the subunit architecture. The (αβ)_4_ architecture is based on the experimentally determined molecular weight of Δ24-SD. Two identical subunits form the homodimeric active site (here the α-subunits are chosen), the other two identical subunits (here the β-subunits) are involved in electron transfer to the external acceptor (A). Electron transfer between the α- and β-subunit requires an acceptor (A); if the latter is missing, no electron transfer to the β-subunit is observed for kinetic reasons and only half of the flavins are reduced. Alternatively, the reduced active site flavin is oxidized directly from (A), and the β-subunit has only a structural function. A (*gray rectangle*), unknown electron acceptor. SQ, semiquinone.

Based on the results obtained from UV/visible absorption and EPR spectroscopy, we propose a radical mechanism for Δ24-SD catalysis (Fig. 8*A*). This mechanism starts with two FMN red SQ radicals, which have been experimentally observed in the as isolated state. One FMN SQ abstracts an H atom from C24, forming a substrate radical intermediate that is stabilized by delocalization *via* the C22 double bond. The second flavin SQ then abstracts a hydrogen atom from C25 giving the Δ22,24 product. Such a mechanism implies that one substrate is bound by two FMN-binding subunits. The impossibility of oxidizing the SQ of both flavins strongly supports a radical-based mechanism, since hydride transfer from the substrate would require a fully oxidized state of at least one of the two flavin cofactors. Surprisingly, even in the presence of an excess of the two-electron donor SDO, only half of the flavins are reduced. To resolve this contradiction, we propose that a α_2_ or β_2_ homodimer rather than a αβ heterodimer forms the SDO-binding catalytic unit (Fig. 8*B*). In such a scenario, one homodimer forms the catalytically active subcomplex, while the second one is involved in electron transfer to an external acceptor. The native molecular weight determined supports an (αβ)_4_ architecture of Δ24-SD, allowing for the proposed arrangement of homodimeric α_2_ or β_2_ subcomplexes, either in a ring or a linear architecture. In the model shown Figure 8*B*, the (αβ)_4_ complex contains only two active sites, and the substrate SDO reduces only the two FMNs of the active site homodimer. Electron transfer from the reduced active site flavin to that of the second subunit occurs only in in the presence of an external electron acceptor for kinetic reasons. Alternatively, the subunits binding the nonactive site flavin may not be involved in electron transfer, but rather have a structural function, *e.g.*, by promoting ring structure formation of the (αβ)_4_ complex.

Phylogenetic analysis revealed that the two subunits of Δ24-SD show high similarity only to uncharacterized putative flavin-dependent oxidoreductases, suggesting that Δ24-SD represents the prototype of a novel class of desaturases. There are no similarities with the functionally related phytoene desaturases (PDS) involved in carotenoid biosynthesis. The two types of membrane-associated PDS introduce either two (PDS type in plants and cyanobacteria) (31) or four (CRTI-type in bacteria) (32) double bonds in conjugation with preexisting ones. Both contain a noncovalently bound active site FAD cofactor, and achieve dehydrogenation *via* a hydride rather than a hydrogen atom transfer mechanism. The only experimentally characterized enzyme with significant similarities to Δ24-SD is limonene DH CtmAB, which catalyzes a water-dependent hydroxylation but not a desaturation reaction (28). Both have a heterodimeric architecture in which each subunit covalently binds a flavin cofactor. A notable difference between Δ24-SD and limonene DH is that in the latter the flavins can be completely oxidized, *e.g.*, by ferrocenium hexafluorophosphate. Consequently, a hydride transfer mechanism *via* a cationic intermediate has been proposed for limonene dehydrogenase, similar to that of the Mo-dependent S26DH, which also catalyzes the hydroxylation of allylic methyl groups. This finding suggests that the two related members of the family of flavin-dependent oxidoreductases acting on methyl groups in the allylic position differ in both, the reaction catalyzed (desaturation *versus* hydroxylation), and the mechanism (radical *versus* cationic intermediates).

## Experimental procedures

### Chemicals and bacterial strain

The chemicals used in this work were of analytical grade and were purchased from Sigma-Aldrich, Merck, Carl Roth, and Carbosynth. *S. denitrificans* Chol-1S^T^ (DSMZ 13999) and *T. aromatica* K172 (DSMZ 6984) were obtained from the Deutsche Sammlung für Mikroorganismen und Zellkulturen.

### Cultivation of *S. denitrificans*

*S. denitrificans* cells were cultivated under denitrifying conditions (100% N_2_ atmosphere) in a phosphate-buffered medium (3 g l^–1^ NaH_2_PO_4_ ∙ 2 H_2_O, 4 g l^–1^ K_2_HPO_4_, and 0.54 g l^–1^ NH_4_Cl, pH 6.9) with 1.5 mM stigmasterol and 5 mM nitrate at 30 °C in a 200-l fermenter. The culture was harvested in the exponential phase using a Cepa Z41 flow centrifuge (Carl Padberg) at 20,000 rpm and 4 °C. Cells were immediately frozen in liquid nitrogen and stored at −70 °C until further use.

### Heterologous production of S26DH_2_ in *T. aromatica* K172

Gene clusters encoding the active site α_5_β_5_γ_5_-, α_6_β_6_γ_6_-, and α_7_β_7_γ_7_-subunits of the Mo-dependent dehydrogenase DH_5_, DH_6,_ and DH_7_, respectively, were heterologously produced as previously described (25). *T. aromatica* cells were cultivated under anaerobic conditions at 30 °C in phosphate-buffered medium (0.6 g l^–1^ NaH_2_PO_4_, 7.3 g l^–1^ K_2_HPO_4_ ∙ 3 H_2_O, 0.54 g l^–1^ NH_4_Cl, pH 7.8) with benzoate and nitrate as electron acceptor in a 1:3.2 mM ratio. The medium was supplemented with 20 μg ml^–1^ gentamycin, 1 mM MgSO_4_ ∙ 7 H_2_O, 0.1 mM CaCl_2_ ∙ H_2_O, vitamin VL-7 solution (33), and trace element solution SL10 (34). Gene expression was induced with 1 mM IPTG (22).

### Synthesis of stigmast-1,4-diene-3-one

SDO was synthesized by enzymatic conversion of commercially purchased stigmasterol (Biosynth) using AcmB produced heterologously in *Escherichia coli* BL21(DE3) cells (25) and cholesterol oxidase from *Streptomyces* sp. (Sigma-Aldrich). The enzymatic reaction mixture contained 50 mM 3-(n-morpholino)propanesulfonic acid (Mops)/KOH buffer at pH 7.0; 6% (w/v) HPCD; 5 mM NAD^+^; 5 mM MgCl_2_; 5 mM K_3_[Fe(CN)_6_]; 2 mM stigmasterol; cholesterol oxidase, catalase, and 10% (v/v) soluble cell extracts from *E. coli* BL21 producing AcmB. Enzymatic conversion was set for 16 h at 30 °C in the dark. Extraction was performed with twice the volume of ethyl acetate and the organic phase was evaporated at 45 °C and 240 mbar. The product was separated and purified by a preparative HPLC (pHPLC) column (XSelect CSH prep C18 5 μM OBD 19 × 250 mm, Waters) using a gradient from 30%-100% water/2-propanol in 44 ml at 2.5 ml min^–1^. The organic solvent of the purified product SDO was evaporated at 135 mbar and 45 °C, dissolved in 2-propanol to a final volume of 100 mM and stored at 6 °C until further use.

### Purification of Δ24-SD from WT *S. denitrificans*

Purification was carried out under aerobic conditions. *S. denitrificans* grown on 1.5 mM stigmasterol cells were resuspended in twice the volume of lysis buffer (20 mM Tris/HCl pH 7.5) and 0.1 mg DNAse. Cells were disrupted twice using a precooled French pressure cell at 1100 psi. Cell debris was removed by ultracentrifugation at 45,000 rpm and 4 °C for 60 min. The supernatant was precipitated with 1.5 M (NH_4_)_2_SO_4_ for 20 min at 8 °C and filtered (20 μm pore size) prior to use. The supernatant was applied to a Butyl-S Sepharose 6 Fast Flow column equilibrated with buffer A_1_ (1.5 M [NH_4_]_2_SO_4_, 20 mM Tris/HCl pH 7.5). Active protein fractions were eluted using a step gradient of 90% to 100% (150 mM and 0 mM (NH_4_)_2_SO_4_) buffer A_2_ (20 mM Tris/HCl pH 7.5) at a flow rate of 3 ml min^–1^ and concentrated using cutoff membranes (30 kDa). Further enrichment was achieved using a HiTrap Capto Q column equilibrated with buffer A_2_. The active protein fraction was eluted using a step gradient of 60% to 90% (300–450 mM KCl) buffer B_1_ (20 mM Tris/HCl, 500 mM KCl pH 7.5). Protein fractions were concentrated to 6 to 16 mg ml^–1^, desalted (PD-10 column) and stored at 8 °C until further use or at −70 °C for long-term storage.

### Determination of MW of Δ24-SD

MW determination was performed by size exclusion chromatography (Superose 6 Increase 10/300 GL). The column was preequilibrated with running buffer (20 mM Tris/HCl pH 7.5, 150 mM KCl). Gel filtration standards consisting of bovine serum albumine (MW = 66.5 kDa), alcohol dehydrogenase (MW = 150 kDa), apoferritin (MW = 443 kDa), and thyroglobulin from bovine thyroid (MW = 670 kDa) were used for calibration.

### Protein identification by mass spectrometry

Samples for liquid chromatography-tandem mass spectrometry liquid chromatography-tandem mass spectrometry analyses were separated by SDS-PAGE. Following visualization of proteins with colloidal Coomassie blue, gel bands were excised and proteins were in-gel digested using trypsin for subsequent mass spectrometry (MS) analysis essentially as described (35). In brief, peptides mixtures were separated on an Ultimate 3000 RSLCnano coupled to a Q-Exactive Plus mass spectrometer (Thermo Fisher Scientific). Peptides were washed and concentrated on μPAC trapping columns and analyzed on a 50 cm μPAC analytical column (both Thermo Fisher Scientific) using a 75 min gradient of solvent A (0.1% formic acid) and solvent B (86% acetonitrile (ACN); 0.1% formic acid). Data dependent acquisition consisted of full MS scans in the range of m/z 370 to 1700; resolution of 70,000 at m/z 400; and fragmentation of the 15 most abundant multiply charged precursor ions by higher energy collisional dissociation recorded with resolution of 35,000. Additionally, UV absorbance was monitored at a wavelength of 370 nm, specific for FAD/FADH_2_. MS raw data were searched using MaxQuant version 2.0.2.0 (36) against the reference proteome protein sequences for *S. denitrificans* from UniProt (release 2022_03, taxonomy id 157592). Carbamidomethylation of cysteine residues was considered as fixed modification, oxidation of methionine, acetylation of protein N termini as variable modifications. Furthermore, flavin cofactors FMN and FAD were allowed as variable modifications at histidine according to Unimod (https://www.unimod.org) id 409 (FMNH, chemical formula H_19_C_17_N_4_O_9_P, monoisotopic mass 454.0890) and id 50 (FAD, chemical formula H_31_C_27_N_9_O_15_P_2_, monoisotopic mass 783.1415), respectively. Proteins were identified with at least one unique peptide and a false discovery rate of 0.01 on both peptide and protein level.

### Enzymatic conversion of SDO to STO and further conversion to (24*E*)-26-hydroxy-stigmasta-1,4,24-triene-3-one (26-OH-STO)

(1) The enzymatic conversion of SDO to STO was tested using a setup containing 2% to 10% (v/v) soluble extracts of *S. denitrificans* or (0.2–0.6 mg ml^–1^) purified enzyme Δ24-SD; 50 mM Mops/KOH pH 7.0; 6% (w/v) HPCD; 1 mM 2,6DCPIP, and 500 μM SDO under aerobic conditions. Depending on the experiment, the reaction mixture was incubated for 10 min to 1440 min at 30 °C and 300 rpm. Kinetic parameters of the purified enzyme were obtained based on the UPLC enzymatic conversion rate using different SDO substrate concentrations until saturation. (2) The setup for the enzymatic conversion of STO to 26-OH-STO contained 50 mM Mops/KOH pH 7.0; 6% (w/v) HPCD; 2.5 mM K_3_[Fe(CN)_6_]; 1 mM STO and 10% soluble extracts of heterologously produced S26DH_2_ from *T. aromatica* under anaerobic conditions.

Both enzyme assays were stopped with four volumes of 2-propanol and centrifuged twice at 4 °C and 14,000 rpm for 15 min. The supernatants were applied to an Acquity UPLC H-class system (Waters) using a CSH C18 column (1.7 μm, 2.1 mm × 100 mm) with a gradient from 5% to 100% ACN in 10 mM aqueous NH_4_OAc at a flow rate of 0.35 ml min^–1^.

### NMR spectroscopy of STO and 26-OH-STO

STO was produced in large scale using the setup of the enzymatic conversion. The 50 ml reaction mixture was stirred and incubated for 24 h in the dark at 30 °C. The product was extracted by adding twice the volume of ethyl acetate. The organic solution was evaporated at 240 mbar and 45 °C. The remaining product was dissolved in 5 ml 2-propanol and purified isocratically *via* pHPLC using a XSelect CSH C18 column (Waters) at 100% ACN. The organic phase of the purified STO fraction was evaporated at 135 mbar and 45 °C. The product was dissolved in 1:10 2-propanol/deionised H_2_O (dH_2_O) and freeze dried at 0.02 mbar and −80 °C overnight. The dried product was dissolved in 600 μl deuterated chloroform (CDCl_3_). 26-OH-STO was produced as described for STO. NMR spectra were measured on a Bruker Avance Neo 400 MHz spectrometer. An internal solvent peak (^1^H NMR: CHCl_3_: 7.26 ppm; ^13^C NMR: CDCl_3_: 77 ppm) was used as a reference. The processed data are noted as follows: chemical shift (δ, ppm), multiplicity (s, singlet; d, doublet; t, triplet; q, quartet; m, multiplet; br. s, broad signal), coupling constant(s) (J, Hz), integration.

The chemical structure of STO was deduced from ^1^H, ^13^C and 2D NMR experiments (for NMR spectra see Figs. S2*A*, S2*B*, S4*A*, S5*A* and S6*A*). ^1^H NMR: δ = 7.04 ppm (1H, d, *J* = 10.14 Hz), 6.23 ppm (2H, m), 6.01 ppm (1H, s), 5.33 ppm (1H, q, ^3^*J* = 15.6 Hz, *J*_trans_ = 8.8 Hz). ^13^C-NMR: δ = 186.4, 169.4, 156.0, 134.1, 132.7, 132.5, 127.9, 127.4, 125.0 ppm.

The chemical structure of (24*E*)-26-OH-STO was deduced from ^1^H, ^13^C and 2D NMR experiments (for NMR spectra, Figs. S3, *A* and *B*, S4*B*, S5*B* and S6*B*). ^1^H NMR: δ = 7.04 ppm (1H, d, *J* = 10.1 Hz), 6.19 ppm (2H, m), 6.05 ppm (1H, s), 5.53 ppm (1H, dd, ^3^*J* = 15.6 Hz, *J*_trans_ = 8.9 Hz), 4.17 ppm (2H, d, *J* = 1.3 Hz). ^13^C-NMR: δ = 186.8, 169.7, 156.2, 137.5, 136.6, 130.1, 127.6, 125.3, 123.9, and 64.3 ppm.

### UV/visible absorption spectroscopy

UV/visible absorption spectroscopy was performed in an anaerobic chamber at 25 °C using a spectrophotometer (UV-1800, Shimadzu) and quartz cuvettes. All solutions were flushed with N_2_ before the experiments. For titration with sodium dithionite (DT), Δ24-SD, based on the protein concentration and using the molecular weight of 120.6 kDa was diluted to a final concentration of 25 μM with 50 mM Mops/KOH pH 7.5 and titrated with freshly prepared 1 mM and 5 mM DT solutions. For substrate reduction, 25 μM Δ24-SD in 50 mM Mops/KOH pH 7.5 buffer were incubated with 25 μM SDO. Spectra were recorded at different time points. The recorded spectra were normalized for their absorption at 800 nm and corrected by the dilution factor. Data were analyzed and plotted using GraphPad Prism 6.04 (GraphPad).

### EPR spectroscopy

Protein samples of 15.5 mg ml^–1^ were used for EPR measurements. CW EPR measurements were carried out at X-band frequency on an EMX-Nano spectrometer (Bruker). The samples were filled into quartz tubes (Ilmasil PS, Qsil) with an inner diameter of 3 mm and shock frozen in liquid nitrogen. The sample volume was adjusted to fill the active height of the resonator completely (25 mm). The temperature was set to 100 K using a nitrogen gas flow cryostat (variable temperature accessory, Bruker). Spectra were recorded with five scans in a range from 3320 to 3520 G with a resolution of 0.3 G. The microwave power was set to 0.316 mW and the modulation amplitude (100 kHz) was set to 3 G.

Pulse EPR spectra were measured on a Q-band ElexSys E580 spectrometer (Bruker) using a Q-band pulse EPR resonator (model EN 5107D2, Bruker). Samples were filled into quartz glass tubes with an inner diameter of 1 mm and shock frozen in liquid nitrogen. Experiments were performed at 80 K using a gas flow cryostat (CF935, Oxford Instruments) and a proportional-integral-derivative temperature controller (ITC4, Oxford Instruments). Microwaves were generated by a super XFT microwave bridge (Bruker) and amplified by a 50 W solid state amplifier (AMPQ34GHz, Bruker). Echo detected field sweep spectra were recorded in a field range from 11,800 to 12,200 G with a resolution of 1 G by using a standard Hahn echo sequence. The length of the π-pulse was set to 32 ns and τ was set to 400 ns. The spectrum was recorded by integrating the full width of the Hahn echo with a 4 ns time increment with a shot repetition time of 1 ms and 50 shots per point.

The radical concentration of the samples was estimated by spin counting. The CW spectra were baseline corrected by subtracting a first order polynomial and double integrated in a range from 3390 to 3480 G. Spin counting was performed with the spin quantification routine of the Xenon software (Bruker). CW-EPR simulations were performed in Matlab (R2022a) using the pepper routine of the EasySpin toolbox (6.0.0-dev.53) (37). The magnetic-field offset was corrected using the spectrum of a carbon fiber with known g-value (2.002644) (38). The *g*-values of the flavin radicals were obtained by nonlinear least squares fitting in esfit (37). The line broadening was defined by unresolved hyperfine couplings (HStrain). Two nitrogen nuclei with axial hyperfine tensors were included in the simulation (N(5) and N(10) of the flavin’s isoalloxazine moiety). For both nitrogens, *A*_⊥_ was set to 0. *A*_||_ was set to 30 MHz and 50 MHz for N(10) and N(5), respectively (39).

## Data availability

All data are available upon request. The protein mass spectrometry data have been deposited to the ProteomeXchange Consortium via the PRIDE (40) partner repository with the dataset identifier PXD048763 and 10.6019/PXD048763.

## Supporting information

This article contains supporting information.

## Conflict of interest

The authors declare that they have no conflicts of interest with the contents of this article.
