# Supplementary material for: Bacterial stigmasterol degradation involving radical flavin delta-24 desaturase and molybdenum-dependent C26 hydroxylase
Source: J Biol Chem. 2024 Mar 30;300(5):107243. doi: 10.1016/j.jbc.2024.107243 (PMC11061730; doi:10.1016/j.jbc.2024.107243)
Supplement: Supplementary Figs. 1–13 and Tables 1–3 [file mmc1.docx]

**Supporting information**

**Bacterial stigmasterol degradation involving a radical flavin delta-24 desaturase and a molybdenum dependent C26 hydroxylase**

Running title: Radical flavin stigmasterol desaturase

Tingyi Zhan^1^, Christian Jacoby^1^, Martin Jede^1^, Bettina Knapp^2^, Sascha Ferlaino^3^, Andreas Günter^4^, Friedel Drepper^2^, Michael Müller^3^, Stefan Weber^4^, and Matthias Boll^1^*

From the

^1^Microbiology, Faculty of Biology, University of Freiburg, Freiburg, Germany; ^2^Biochemistry and Functional Proteomics, Faculty of Biology, University of Freiburg, Freiburg, Germany; ^3^Institute of Pharmaceutical Sciences, University of Freiburg, Freiburg, Germany; ^4^Institute of Physical Chemistry, University of Freiburg, Freiburg, Germany.

­­­­­­­­­­

Supplementary Figs 1-13

Supplementary Tables 1-3

## Supplementary figures

**Figure S1. Growth curve of S. denitrificans grown with 1.5 mM stigmasterol under denitrifying conditions in a 200-L-fermenter.** Growth was monitored by measuring optical density at 578 nm (OD_578nm_), pH and NaNO_3_ consumption. The arrows indicate supplementation with 5 mM NaNO_3_. Black dots, OD_578nm_; grey squares, pH.


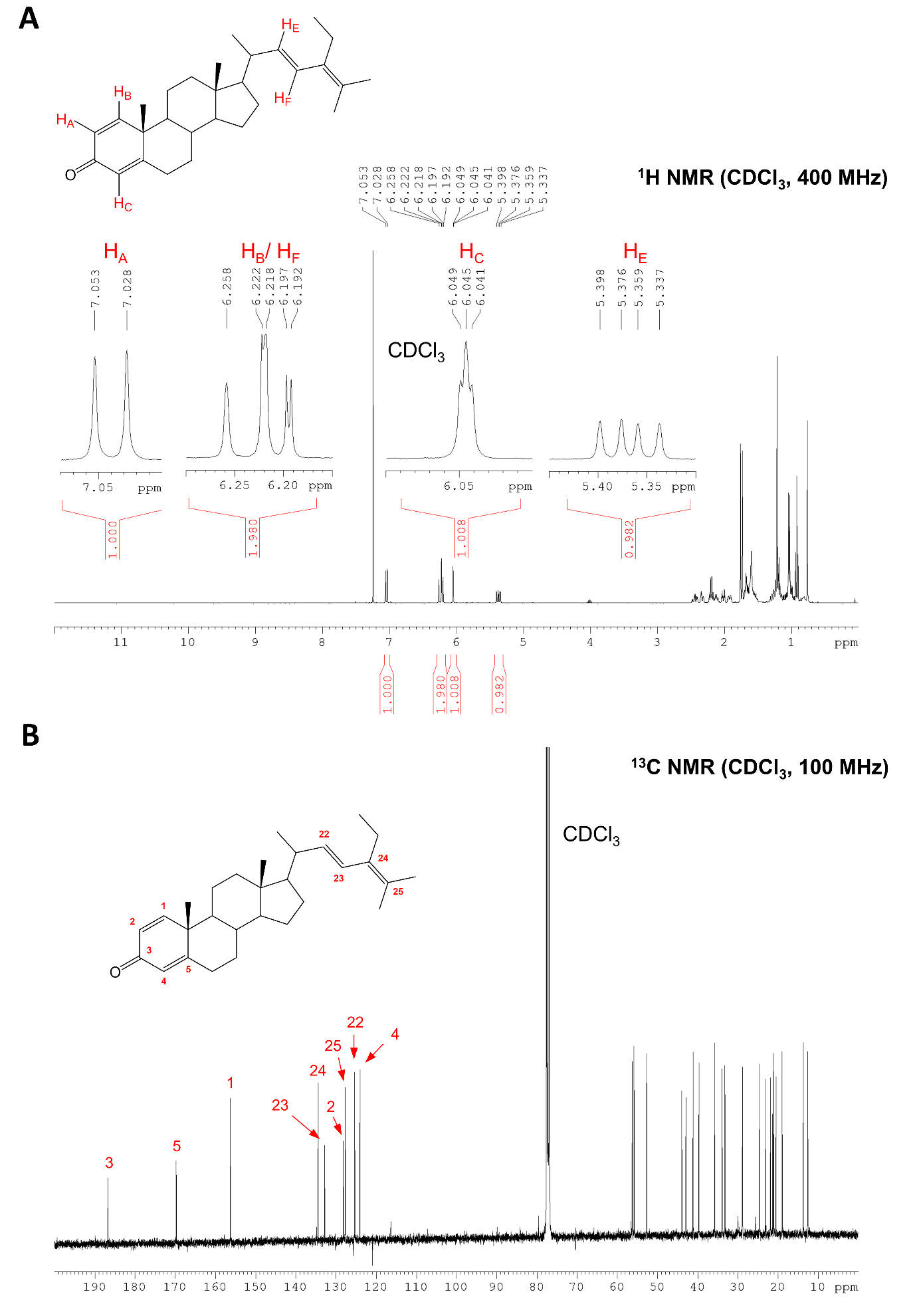


**Figure S2. A,** ^1^H NMR spectra (400 MHz, CDCl_3_) of the product stigmasta-1,4,24-triene-3-one obtained by enriched Δ24-SD from wild type S. denitrificans; **B,** ^13^C NMR spectra (100 MHz, CDCl_3_) of the product stigmasta-1,4,24-triene-3-one obtained by enriched Δ24-SD from wild type S. denitrificans.

**
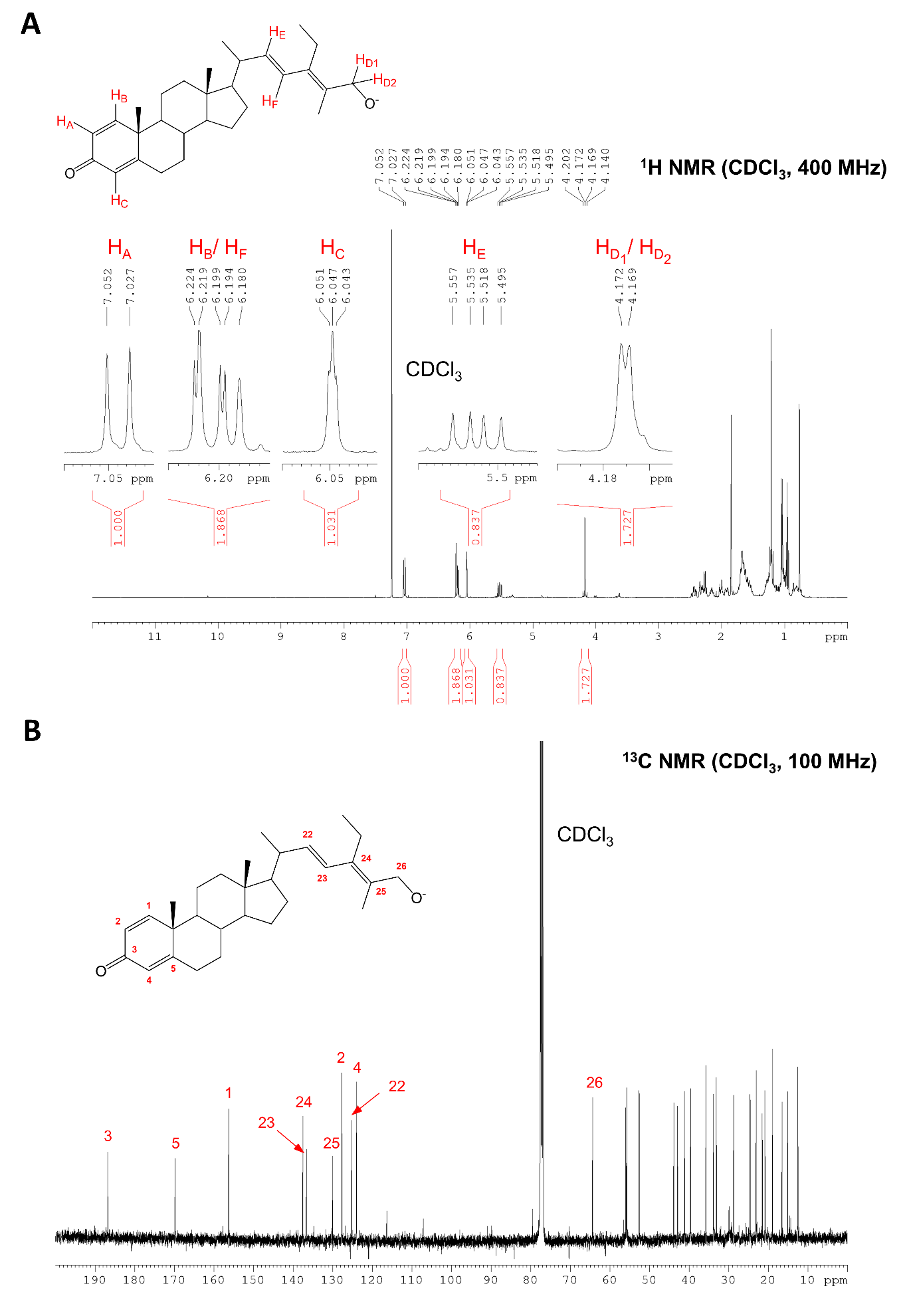
**

**Figure S3. A**, ^1^H NMR spectra (400 MHz, CDCl_3_) of the product (24E)-26-hydroxy-stigmasta-1,4,24-triene-3-one obtained by recombinant S26DH_2_ by T. aromatica extracts; **B,** ^13^C NMR spectra (100 MHz, CDCl_3_) of the product (24E)-26-hydroxy-stigmasta-1,4,24-triene-3-one obtained by recombinant S26DH_2_ by T. aromatica extracts.


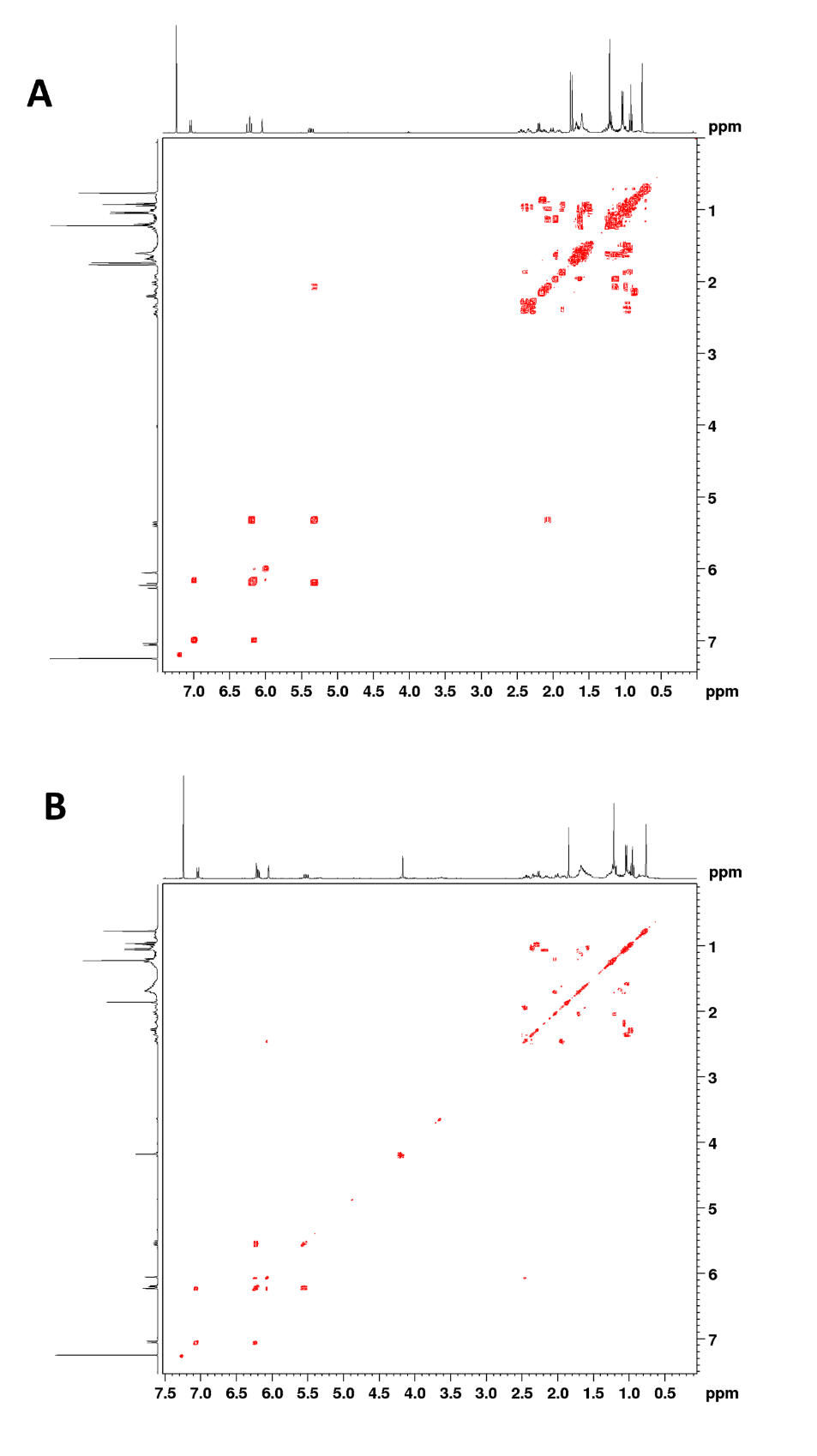


**Figure S4. A**, HH COSY NMR spectra of the product stigmasta-1,4,24-triene-3-one obtained by enriched Δ24-SD from wild type S. denitrificans; **B**, HH COSY NMR spectra of the product (24E)-26-hydroxy-stigmasta-1,4,24-triene-3-one obtained by recombinant S26DH_2_ produced in T. aromatica extracts.


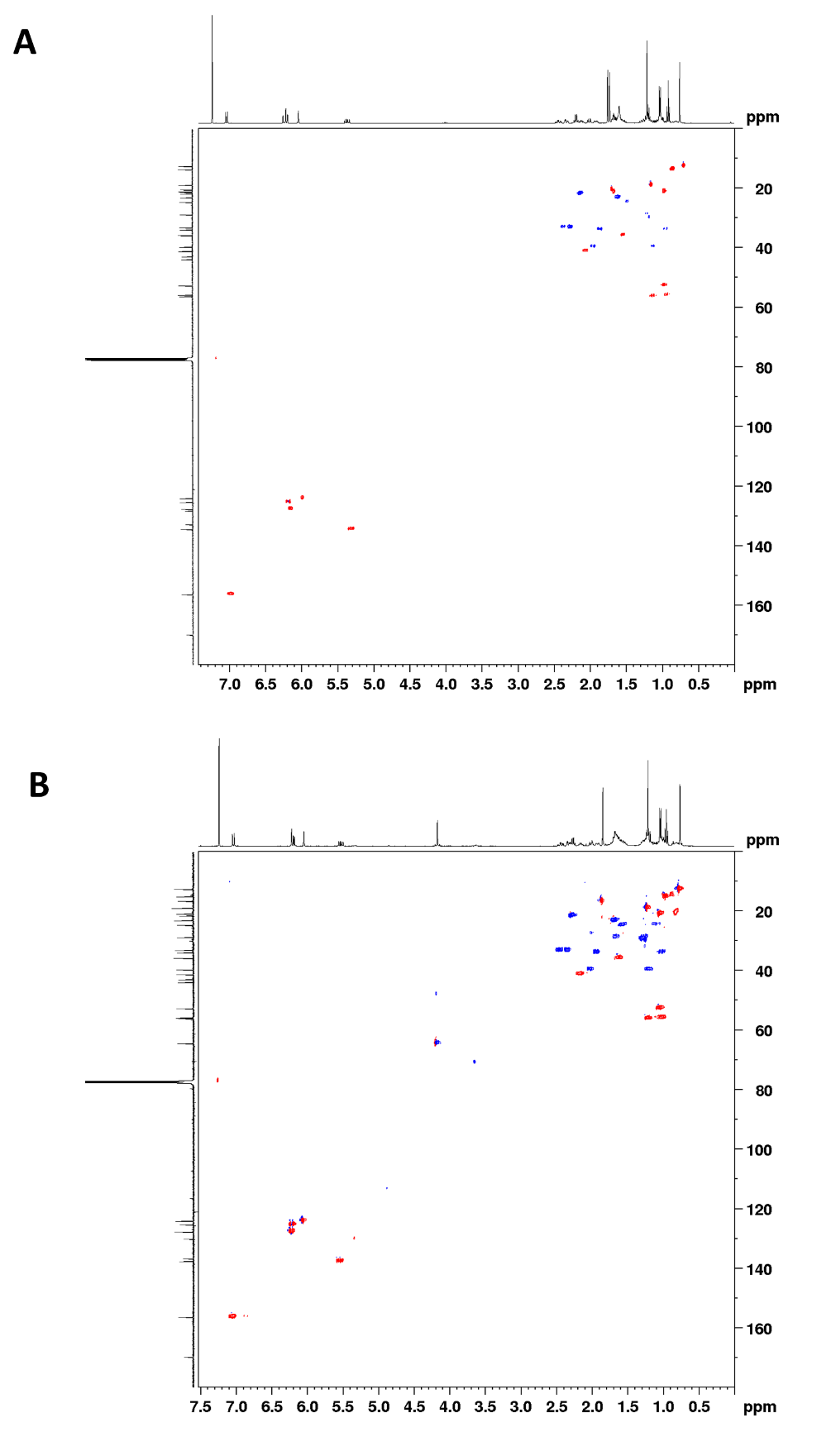


**Figure S5. A,** HSQC NMR spectra of the product stigmasta-1,4,24-triene-3-one obtained by enriched Δ24-SD from wild type S. denitrificans; **B**, HSQC NMR spectra of the product (24E)-26-hydroxy-stigmasta-1,4,24-triene-3-one obtained by recombinant S26DH_2_ by T. aromatica extracts.

**
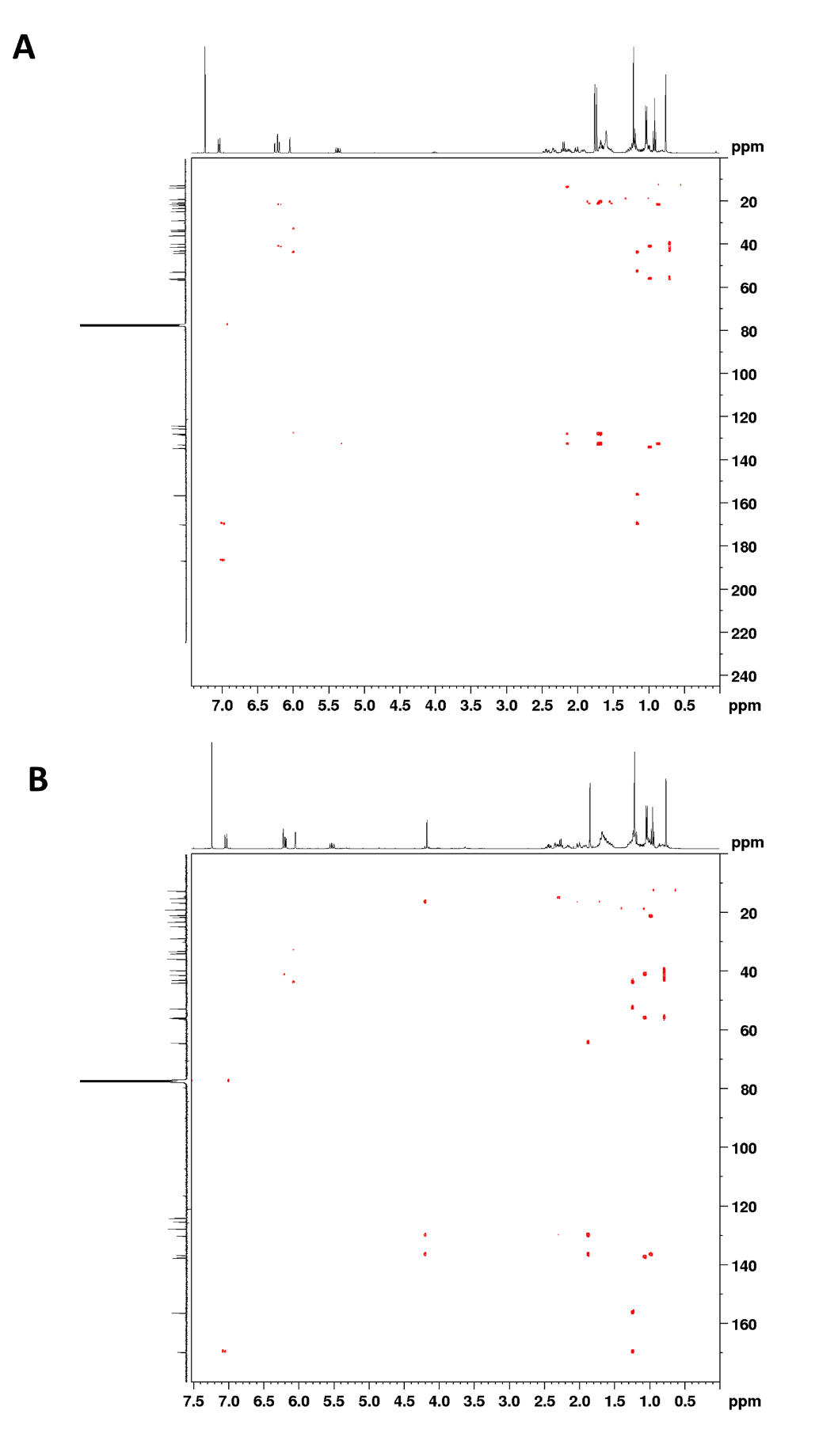
**

**Figure S6. A,** HMBC NMR spectra of the product stigmasta-1,4,24-triene-3-one obtained by enriched Δ24-SD from wild type S. denitrificans. **B,**  HMBC NMR spectra of the product (24E)-26-hydroxy-stigmasta-1,4,24-triene-3-one obtained by recombinant S26DH_2_ by T. aromatica extracts.


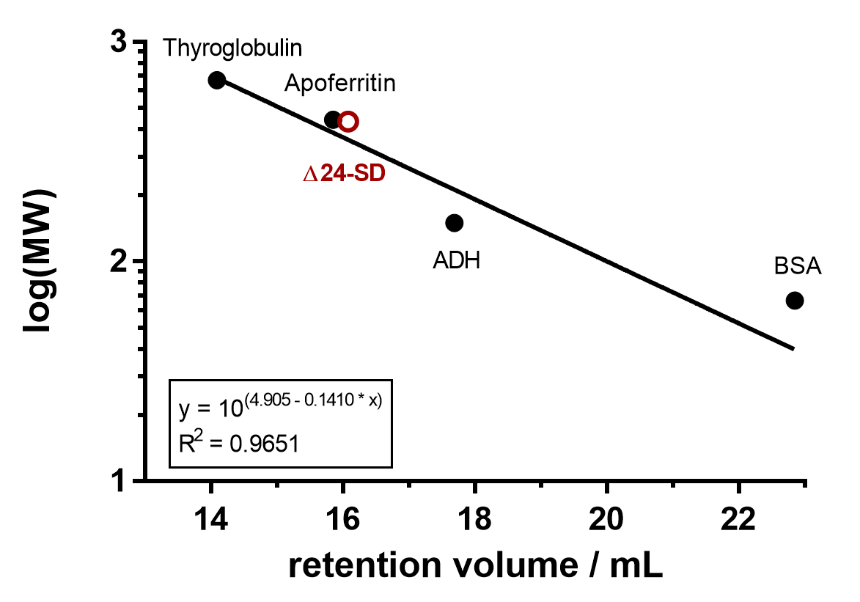


**Figure S7. Molecular weight determination of Δ24-SD.** The molecular weight was determined by size exclusion chromatography (Superose™ 6 Increase 10/300 GL) using commercially available gel filtration standards. Molecular weight (MW) in kDa: thyroglobulin from bovine thyroid = 670, apoferritin = 443, alcohol dehydrogenase (ADH) = 150, bovine serum albumin (BSA) = 66.5. Retention volume in mL: thyroglobulin from bovine thyroid = 14.1, apoferritin = 15.85, alcohol dehydrogenase (ADH) = 17.69, bovine serum albumin (BSA) = 22.85, Δ24-SD = 16.01.


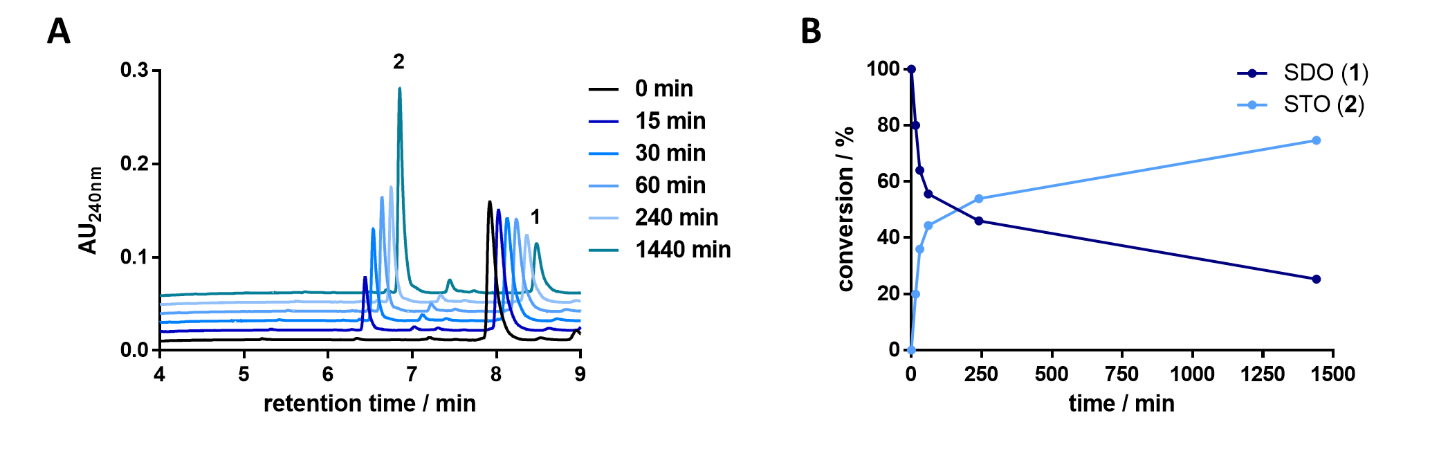


**Figure S8. Conversion of stigmast-1,4-diene-3-one (SDO) (1) to stigmasta-1,4,24-triene-3-one (STO) (2) by enriched Δ24-SD from S. denitrificans in the presence of DCPIP as artificial electron acceptor. A**, UPLC elution chromatogram showing the time-dependent conversion of 0.5 mM SDO to STO by enriched Δ24-SD. **B**, Representative plot showing the quantitative analysis of the ULPC-based assay.


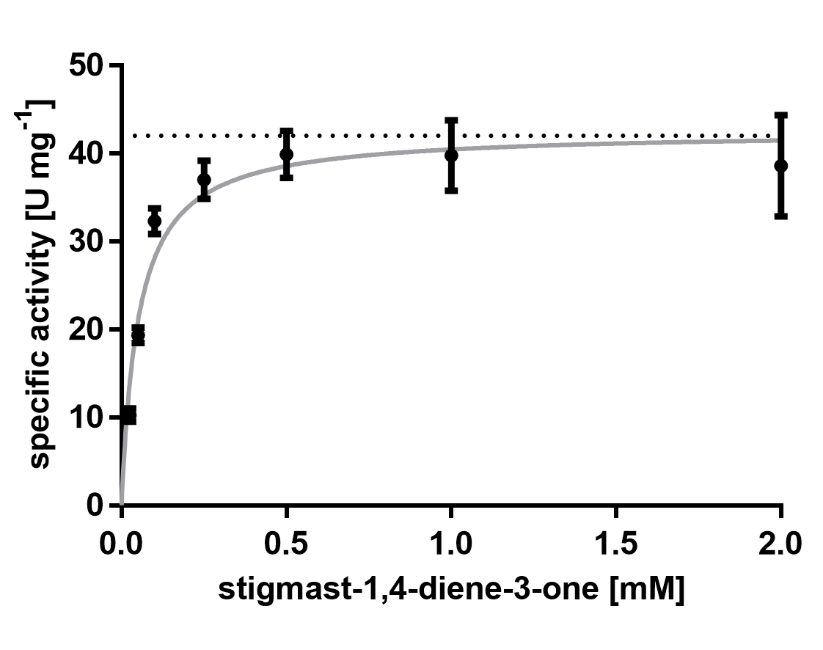


**Figure S9. Fit of kinetic data obtained with Δ24-SD to a Michaelis-Menten curve.** The specific activity was plotted against the substrate concentration. Michealis-Menten fit is shown in grey. The error bars represent standard deviations of the mean value of three independent replicates.


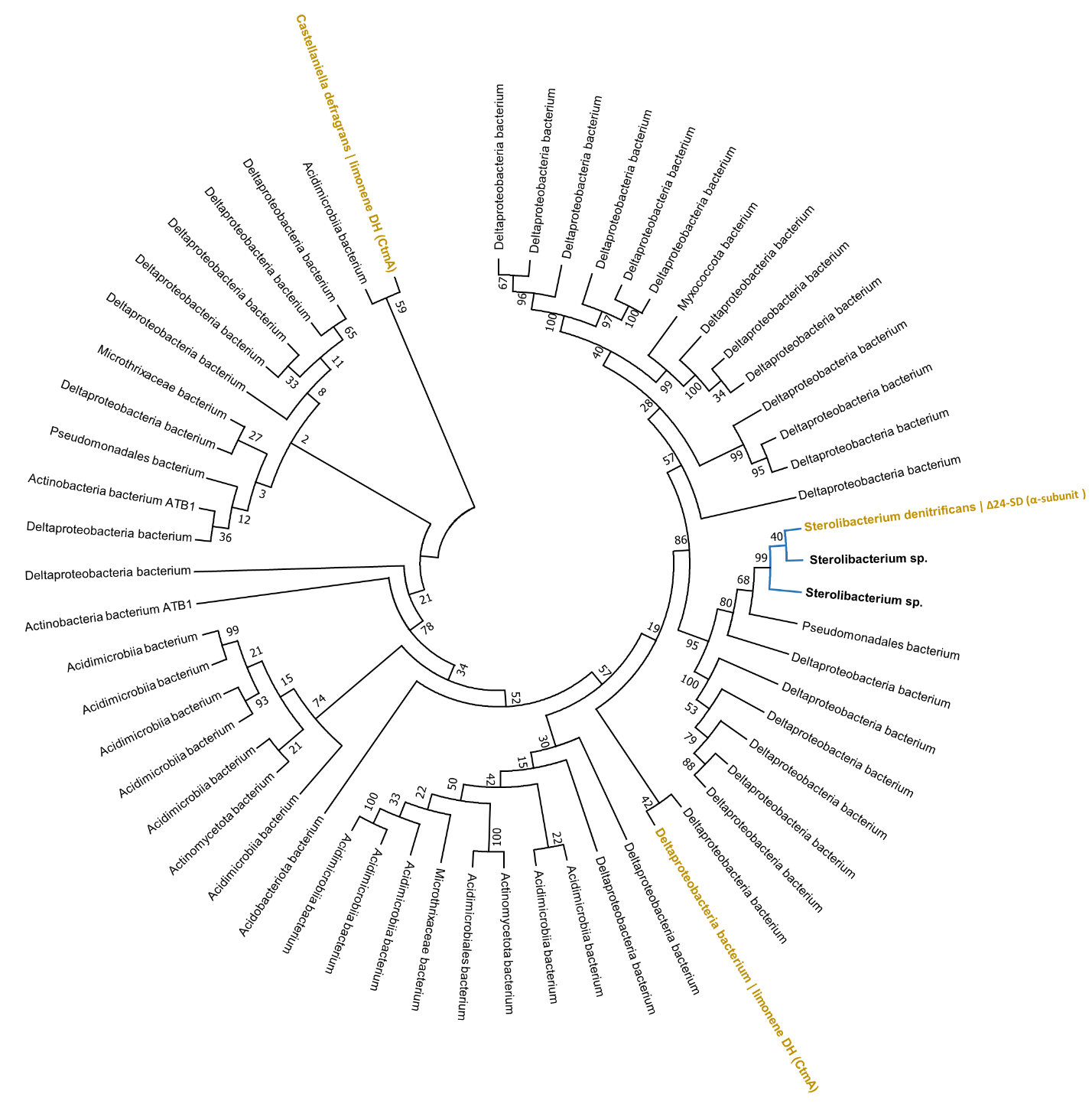


**Figure S10. Phylogenetic tree of the α-subunit of Δ24-SD.** The phylogenetic tree was constructed using the Maximum Likelihood method based on the Jones-Taylor-Thornton substitution model (1000 bootstrap values). Data collection (non-redundant NCBI database) criteria included a sequence identity threshold of >50% an e-value of <1e^–50^. The CtmA sequence of limonene dehydrogenase (DH) from Castellaniella defragrans was included separately. The α-subunit of Δ24-SD from S. denitrificans and the CtmA subunit of limonene DH from Castellaniella defragrans and Deltaproteobacterium are highlighted in yellow. The most similar sequences to the α-subunit of Δ24-SD are highlighted in bold and the nodes in blue. Unless otherwise stated, the protein sequences are annotated as NAD(P)/FAD-dependent oxidoreductases.


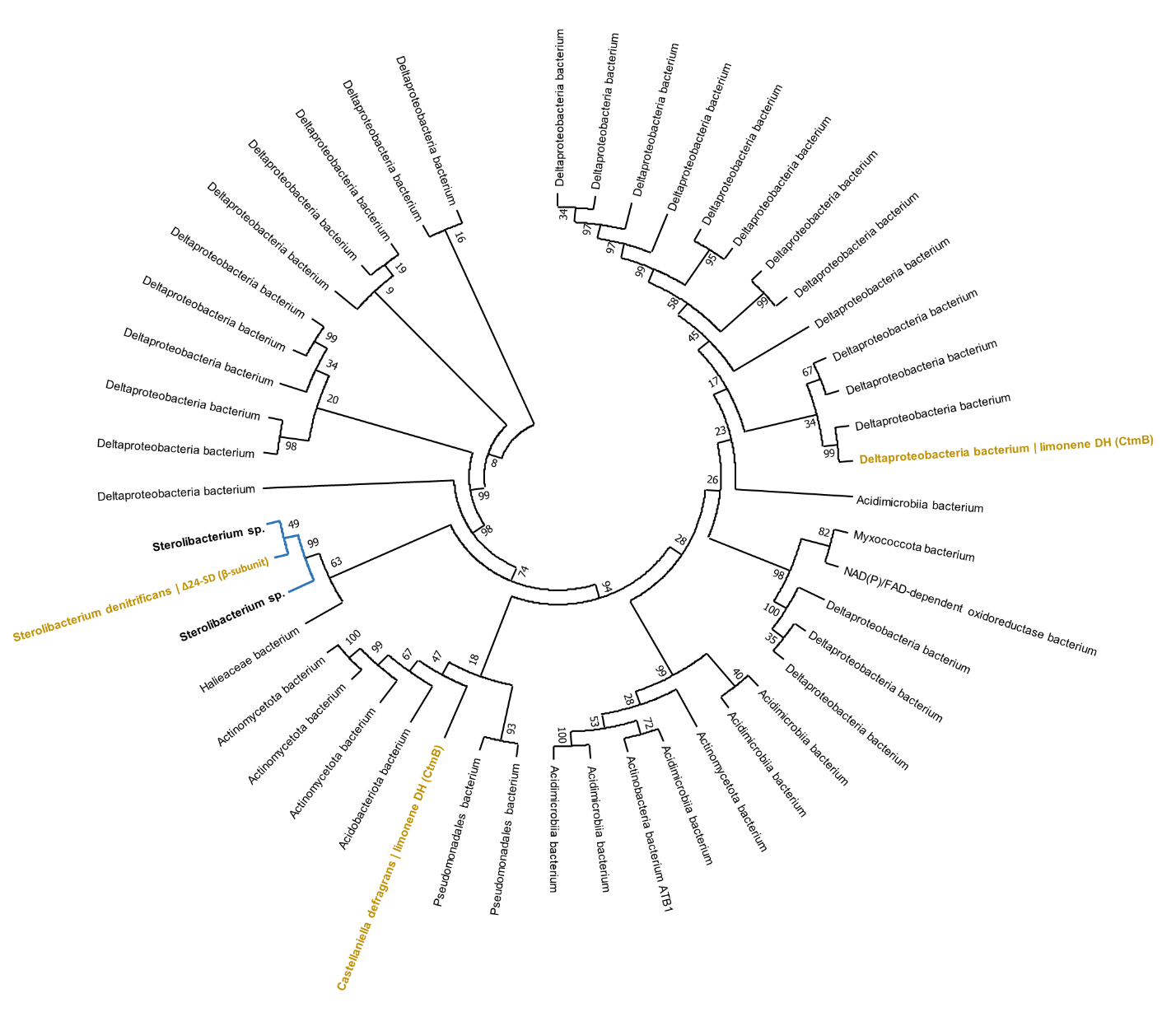


**Figure S11. Phylogenetic tree of the β-subunit of Δ24-SD.** The phylogenetic tree was constructed using the Maximum Likelihood method based on the Jones-Taylor-Thornton substitution model (1000 bootstrap values). Data collection (non-redundant NCBI database) criteria included a sequence identity threshold of >50% and an e-value of <1e^–50^. The CtmB sequence of limonene dehydrogenase (DH) from Castellaniella defragrans was included separately. The β-subunit of Δ24-SD from S. denitrificans and the CtmB subunit of limonene DH from Castellaniella defragrans and Deltaproteobacterium are highlighted in yellow. The most similar sequences to the β-subunit of Δ24-SD are highlighted in bold and the nodes in blue. Unless otherwise stated, the protein sequences are annotated as NAD(P)/FAD-dependent oxidoreductases.


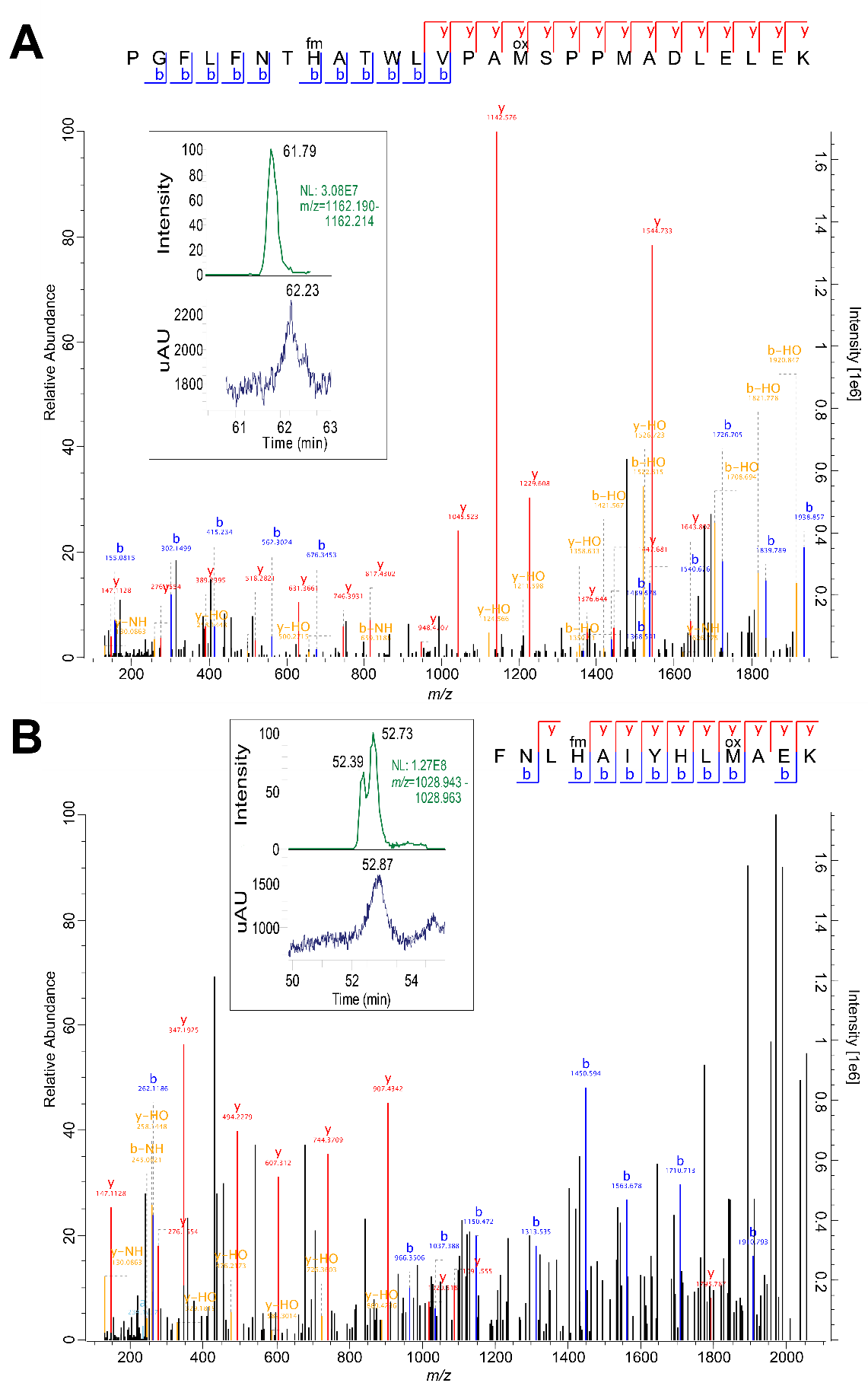


**Figure S12. Representative MS/MS spectra identifying peptides with FMN cofactors bound to His57 in α (A) and His58 in β subunit (B) of Δ24-SD**. Insets show extracted ion and UV chromatograms depicting nano-LC elution of the peptide ion precursors. The coverage of both peptide sequences by fragment ions (b and y) is shown schematically.


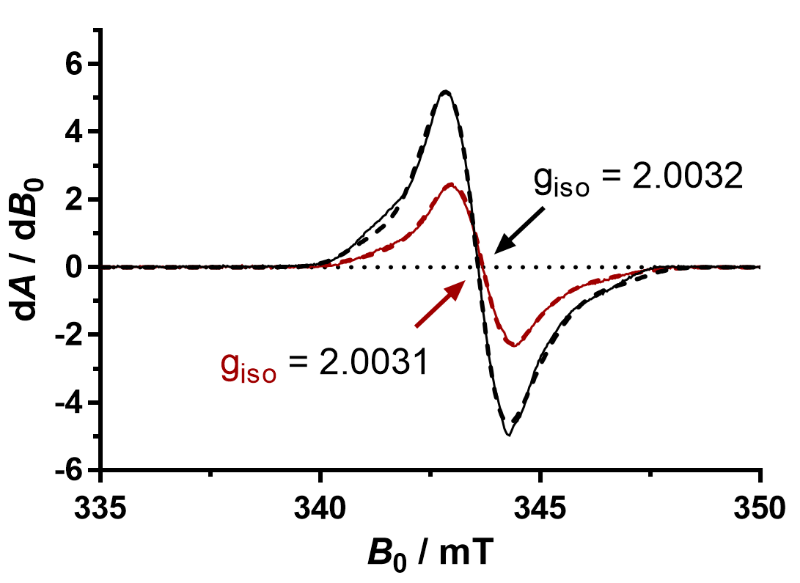


**Figure S13. Simulated (dashed line) and experimental (solid line) continuous-wave EPR spectra of Δ24-SD as isolated (black) and with stoichiometric equivalents of stigmasta-1,4-diene-3-one (SDO) (red) at X-band frequency (9.63 GHz).** The simulations were calculated by non-linear least squares fitting to the experimenta data using the g-value of carbon fiber g = 2.002644. Two nitrogen nuclei with axial hyperfine tensors were included in the simulation. A_⊥_= 0 MHz for both nuclei, A_||_(N(5)) = 30 MHz and A_||_(N(10)) = 50 MHz.

## Supplementary Table

**Table S1 ESI-QTOF-MS analysis**. Proposed intermediates during the degradation of the unsaturated side of stigmasterol. Chemical structure, sum formula, predicted and detected masses and its deviation are given.

| **Compound ID** | **Chemical structure** | **Sum formula** | **Predicted mass [Da]** | **Detected**  **m/z [Da]^a^** | **Deviation**  **(ΔmDa)** |
| --- | --- | --- | --- | --- | --- |
| Stigmast-1,4-diene-3-one (SDO) | 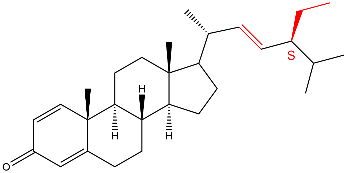 | C_29_H_44_O | 409.3470 | 409.3473 | 0.3 |
| Stigmasta-1,4,24-triene-3-one (STO) | 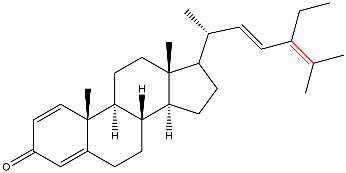 | C_29_H_42_O | 407.3314 | 407.3320 | 0.6 |
| (24*E*)-26-hydroxy-stigmasta-1,4,24-triene-3-one  (26-OH-STO) | 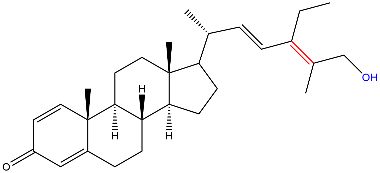 | C_29_H_42_O_2_ | 423.3263 | 423.3249 | 1.4 |
| STO-26-al | 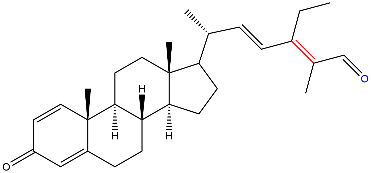 | C_29_H_40_O_2_ | 421.3107 | 421.3101 | 0.6 |

^a^Determined as [M + H]^+^ ions

**Table S2 Amino acid sequence identities of Δ24-SD from S. denitrificans.**

| **Δ24-SD** | **Amino acid sequence** | **Mass deduced from amino acid sequence [kDa]** | ***S. denitrificans* [SDENChol_XXXXX]** | **NCBI accession number** |
| --- | --- | --- | --- | --- |
| α-subunit | MKNYDVIIIGGGINGLTTACYLQKAGLSVGVFEARGQCGAHCDTIELGRPGFLFNTHATWLVPAMSPPMADLELE  KFNLELLGTDVIFAMPHADGTNTVQALDPGITMASIARHSEKDAALLGRIMEFGMMHMQEALEINGQMQYSRP  TMALAERIAKLNDGLCKHLGLPIDGDDVMRMTGFELLELVYESEKVRLVPGTLGEYTGQWPLNRRVAPTVLGLCG  MNPMAVHTAKGGSHALNHALVRCFVKHGGEIWTTCPVSKIIVENGRAVGIKLSEDALMPGEEIRAKNIVSNLTLTP  TFTKLLGEEVIGPDWMRRIKYFNYDDPQLLGMYYAMKDAPVFKSAEYDPAIQRCWVGYFGPDSIDDVRNAQSQV  MAGIMPTQTMGGWFNPTLADPSQAPAGHHVVSTWITVPPSPRKWGNKTLNGWSSWREGFGEALADTMDDL  YEKMAPGFKDLIIERHINTPMDQENSNPSAVRGNMIGGSAIPEQAGENRPLPGVCVNGASRTFIPGLYLSNSIHPFG  ATHLASGYIAAVELAEDLGCRNQSWWISQPFEWFMGNLGNIPLNAGVADKWKV* | 63 | 21263 | WP_067170564 |
| β-subunit | MSNETKFDAIVIGGGPNGLLAGAYLAKTGHKVVLFERRHETGGGLNTDEYFGFRFNLHAIYHLMAEKMPAWKDL  DLANFGVRYLYPHVVAAFPFKDGSSLIFTRDVKETAESIAQFSKEDAAAYLAMWDEFQPMLDDYLIPMTYELPKPA  LDQLAEFGETEVGAKLAEISELSCLEVIDHYGFTHPRVRMALLSFPAMWGIHLADPLGFLYPLYLGRMLDAAFVKG  GSHRLSSGMYRVFVKNGGTVIDENEVTRILVEDNRVTGVELQDGRRFMADAVVSTLNPVQTFKQLLPKDDVPYA  LHQAVDNWRWEERSFFGLHLGIDGGVRYNAQDPRVNEAMTVFIGLETEDELLDHLDRVDGKTTNGPEWLHIAQ  PTTFDKTMAPPNHSVIRAEAVVAYDPEWRARTPAFAAGCLDLIKQYATVDKVVQQRTVSPIDIEEKLTTMHRGSY  KHGAYSTLQLGYLRPNDLCSCSETPIDGLFLGGASMYPGGMILGGPGYLAAQVTNDYLGEPVKK* | 57 | 21262 | WP_067170561 |

**Table S3 Relative activity of enriched Δ24-SD from S. denitrificans with various steroid substrates containing modifications in the isoprenoid side chain.** The enzyme assays contained 1 mM DCPIP and 6% (w/v) HPCD; the specific activity was determined by UPLC analysis.

| **Substrate** | **Chemical structure** | **Relative activity**  **of Δ24-SD*** |
| --- | --- | --- |
| stigmast-1,4-diene-3-one | 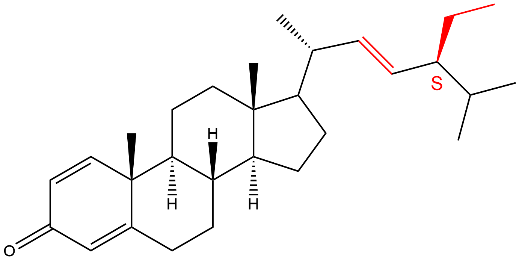 | 100% |
| β-sitost-1,4-diene-3-one | 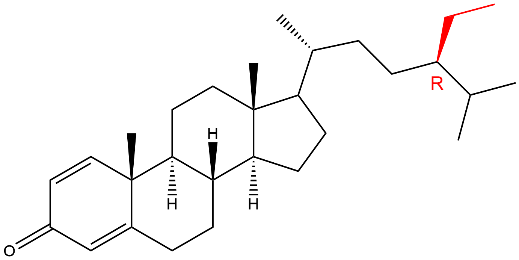 | <1% |
| ergosterol | 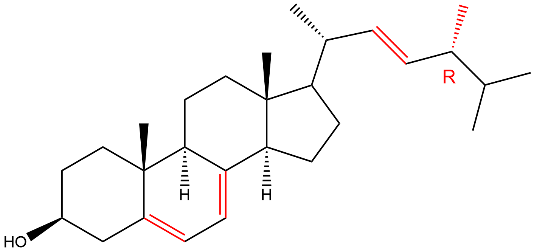 | <1% |
| brassicast-1,4-diene-3-one | 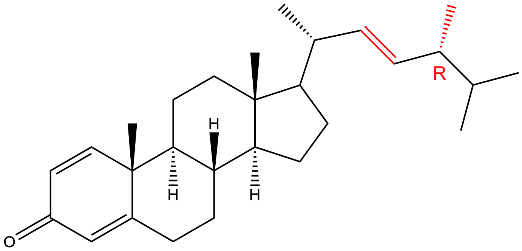 | <1% |
| fucost-1,4-diene-3-one | 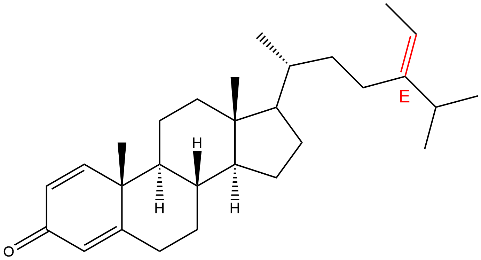 | <1% |

^*^100% activity corresponds to 46.5 mU mg^–1^ with stigmast-1,4,diene-3-one as substrate using enriched

Δ24-SD from *S. denitrificans*.
